# Allelic variation in class I HLA determines CD8^+^ T cell repertoire shape and cross-reactive memory responses to SARS-CoV-2

**DOI:** 10.1126/sciimmunol.abk3070

**Published:** 2021-11-18

**Authors:** Joshua M. Francis, Del Leistritz-Edwards, Augustine Dunn, Christina Tarr, Jesse Lehman, Conor Dempsey, Andrew Hamel, Violeta Rayon, Gang Liu, Yuntong Wang, Marcos Wille, Melissa Durkin, Kane Hadley, Aswathy Sheena, Benjamin Roscoe, Mark Ng, Graham Rockwell, Margaret Manto, Elizabeth Gienger, Joshua Nickerson, MGH COVID-19 Collection and Processing Team, Amir Moarefi, Michael Noble, Thomas Malia, Philip D. Bardwell, William Gordon, Joanna Swain, Mojca Skoberne, Karsten Sauer, Tim Harris, Ananda W. Goldrath, Alex K. Shalek, Anthony J. Coyle, Christophe Benoist, Daniel C. Pregibon, Nikolaus Jilg, Jonathan Li, Alex Rosenthal, Colline Wong, George Daley, David Golan, Howard Heller, Arlene Sharpe, Betelihem A. Abayneh, Patrick Allen, Diane Antille, Katrina Armstrong, Siobhan Boyce, Joan Braley, Karen Branch, Katherine Broderick, Julia Carney, Andrew Chan, Susan Davidson, Michael Dougan, David Drew, Ashley Elliman, Keith Flaherty, Jeanne Flannery, Pamela Forde, Elise Gettings, Amanda Griffin, Sheila Grimmel, Kathleen Grinke, Kathryn Hall, Meg Healy, Deborah Henault, Grace Holland, Chantal Kayitesi, Vlasta LaValle, Yuting Lu, Sarah Luthern, Jordan Marchewka Schneider, Brittani Martino, Roseann McNamara, Christian Nambu, Susan Nelson, Marjorie Noone, Christine Ommerborn, Lois Chris Pacheco, Nicole Phan, Falisha A Porto, Edward Ryan, Kathleen Selleck, Sue Slaughenhaupt, Kimberly Smith Sheppard, Elizabeth Suschana, Vivine Wilson, Mary Carrington, Maureen Martin, Yuko Yuki, Galit Alter, Alejandro Balazs, Julia Bals, Max Barbash, Yannic Bartsch, Julie Boucau, Mary Carrington, Josh Chevalier, Fatema Chowdhury, Edward DeMers, Kevin Einkauf, Jon Fallon, Liz Fedirko, Kelsey Finn, Pilar Garcia-Broncano, Musie S. Ghebremichael, Ciputra Hartana, Chenyang Jiang, Kelly Judge, Paulina Kaplonek, Marshall Karpell, Peggy Lai, Evan C. Lam, Kristina Lefteri, Xiaodong Lian, Mathias Lichterfeld, Daniel Lingwood, Hang Liu, Jinqing Liu, Natasha Ly, Zachary Manickas Hill, Ashlin Michell, Ilan Millstrom, Noah Miranda, Claire O'Callaghan, Matthew Osborn, Shiv Pillai, Yelizaveta Rassadkina, Alexandra Reissis, Francis Ruzicka, Kyra Seiger, Libera Sessa, Christianne Sharr, Sally Shin, Nishant Singh, Weiwei Sun, Xiaoming Sun, Hannah Ticheli, Alicja Trocha-Piechocka, Bruce Walker, Daniel Worrall, Xu G. Yu, Alex Zhu

**Affiliations:** ^1^ Repertoire Immune Medicines; Cambridge, MA, USA; ^2^ Massachusetts General Hospital, Harvard Medical School, Boston, MA, USA; ^3^ Division of Biological Sciences, Molecular Biology Section, University of California, San Diego; San Diego, CA, USA; ^4^ Institute for Medical Engineering and Science, Department of Chemistry and Koch Institute for Integrative Cancer Research, Massachusetts Institute of Technology; Cambridge, MA, USA; ^5^ Broad Institute of MIT and Harvard; Cambridge, MA, USA; ^6^ Ragon Institute of MGH, MIT, and Harvard; Cambridge, MA, USA; ^7^ Department of Immunology, Harvard Medical School; Boston, MA, USA

## Abstract

Effective presentation of antigens by HLA class I molecules to CD8^+^ T cells is required for viral elimination and generation of long-term immunological memory. In this study, we applied a single-cell, multi-omic technology to generate a unified ex vivo characterization of the CD8^+^ T cell response to SARS-CoV-2 across 4 major HLA class I alleles. We found that HLA genotype conditions key features of epitope specificity, TCR α/β sequence diversity, and the utilization of pre-existing SARS-CoV-2 reactive memory T cell pools. Single-cell transcriptomics revealed functionally diverse T cell phenotypes of SARS-CoV-2-reactive T cells, associated with both disease stage and epitope specificity. Our results show that HLA variations significantly influence the CD8^+^ T cell repertoire shape and utilization of immune recall upon SARS-CoV-2 infection.

## Teaser

We perform a unified characterization of CD8^+^ T cell responses to SARS-CoV-2, revealing pre-existing reactivity shaped by HLA genotype.

## INTRODUCTION

Elicitation of a robust and durable neutralizing antibody response following immunization of large sections of the population with approved SARS-CoV-2 vaccines is limiting viral transmission and decreasing mortality, providing hope that the global threat from the COVID-19 pandemic is diminishing. However, the appearance of new viral variants warrants continued vigilance. A more complete understanding of the underlying cellular mechanisms that regulate host immunity and contribute to long term protection is required. Infection with SARS-CoV-2 leads to an upper respiratory tract infection, which can be benign or even asymptomatic. If not controlled by the immune response, it can evolve into a lethal pneumonia with immunopathology due to excessive amplification of the innate inflammatory response, complicated by several extra-respiratory manifestations (*1*). While humoral responses play an important role in immunological control of infection, the generation of effective cellular immunity and expansion of cytotoxic CD8^+^ memory T cells is also required to eliminate virally infected cells as shown from the earlier SARS-CoV-1 epidemic, even in the absence of seroconversion (*2*–*7*).

Several recent studies have focused on the discovery of relevant SARS-CoV-2 epitopes in both CD4^+^ and CD8^+^ T cell responses, leveraging *in silico* predictions, stimulation/expansion with peptide pools (*8*–*18*), tetramer binding (*19*, *20*), and analysis of presentation in vitro (*21*). Collectively, these studies identified a number of immunodominant epitopes derived from across the viral proteome including structural and non-structural proteins in canonical (*8*–*20*) and non-canonical open reading frames (*21*). Interestingly, some of these specificities were also detected in uninfected individuals, suggesting potential cross-reactivity from endemic human coronaviruses (HCoV) to which the population is routinely exposed (*22*), though a direct connection to pre-existing memory cells has not been established.

The breadth and nature of the cellular immune response to SARS-CoV-2 infection is driven by diversity in both T cell receptor (TCR) repertoire and human leukocyte antigen (HLA) genetics. Mammalian cells express up to six different HLA class I alleles that shape antigen presentation in disease, and allelic diversity has been associated with both disease susceptibility and outcome of viral infections (*23*, *24*). There are divergent reports regarding HLA polymorphism and COVID-19 incidence and severity, although the major genome-wide association studies clearly show no dominant effect of the locus (*25*–*29*). Together with genetic influences on HLA-associated antigen presentation, the clonal selection of TCRs that compose an individual’s repertoire contributes to the nature and dynamics of the antiviral response, including cellular cytotoxicity and memory formation. Interestingly, despite a potential TCR diversity of 10^15^ (*30*), several studies have described “public” T cell responses in COVID-19, where complementarity-determining region (CDR) sequences are conserved within and across individuals (*18*, *31*). The extent to which TCR diversity, especially in the context of epitope specificity restricted to HLA, contributes to response is not well understood.

Here, we leverage an assay technology to elucidate, at single-cell resolution, the connection between T cell specificity, HLA variation, conserved features of paired α/β TCR repertoires, and cellular phenotype observed in CD8^+^ T cell responses to SARS-CoV-2 infection. We profiled 96,909,416 CD8^+^ T cells ex vivo across 78 samples from acute, convalescent, or unexposed individuals, and identified T cell specificity to 648 epitopes presented by four HLA alleles across the SARS-CoV-2 proteome, few of which are implicated by the current variants of concern. Estimated frequencies of epitope-specific CD8^+^ T cells observed in convalescent patients had a mean value of 0.01% and maximum around 1% of the total CD8^+^ T cell population. We observed that TCR repertoires were surprisingly public in nature, though we found a high degree of pre-existing immunity associated with a clonally diverse response to HLA-B*07:02, which can efficiently present homologous epitopes from SARS-CoV-2 and HCoVs. Transcriptomic analysis and functional validation confirmed a central memory phenotype and TCR cross-reactivity in unexposed individuals with HLA-B*07:02. Our data suggest an association between HLA genotype and the CD8^+^ T cell response to SARS-CoV-2, which may have important implications for understanding herd immunity and elements of vaccine design that are likely to confer long-term immunity to protect against SARS-CoV-2 variants and related viral pathogens.

## RESULTS

### Direct ex-vivo detection and decoding of SARS-CoV-2-specific CD8^+^ T cells

**Fig. 1.**
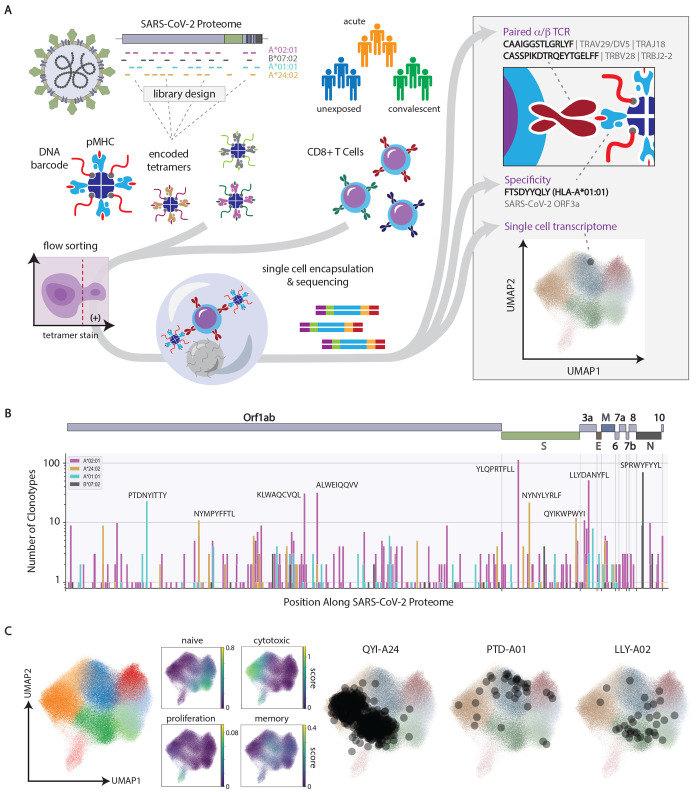
Overview of the experimental approach to decode the CD8^+^ T cell response to SARS-CoV-2. (A) Schematic of the method where encoded tetramer libraries, designed independently for each HLA allele to span the entire SARS-CoV-2-proteome, are used to stain enriched CD8^+^ cells from subject PBMCs, which are then sorted and subjected to single-cell sequencing (left). Using this approach, TCR sequence, peptide/HLA specificity and transcriptomic features are simultaneously acquired for each cell (right). (B) Clonotype specificity detected by HLA allele and epitope across the SARS-CoV-2 proteome in COVID-19 patient samples (n=61). A scheme of the viral ORF structure is shown at the top. Bar colors denote HLA allele. Amino acid sequences of epitopes recognized by the largest number of T cell clonotypes are shown next to the corresponding bar. (C) Single-cell transcriptomic analysis showing global uniform manifold approximation and projection (UMAP) clustering, scoring by functional gene set, and projections onto the transcriptomic UMAP for T cells with specificity toward select epitopes in convalescent individuals (n=33 samples). QYI-A24, PTD-A01, and LLY-A02 correspond to QYIKWPWYI in A*24:02, PTDNYITTY in A*01:01, and LLYDANYFL in A*02:01, respectively.

We leveraged single-cell RNA-sequencing with DNA-encoded peptide-HLA tetramers to characterize CD8^+^ T cell responses to SARS-CoV-2 across multiple Class I alleles in subjects with varying degrees of disease severity. The technology simultaneously determines the specificity of paired α/β TCR sequences for HLA-restricted epitopes, provides transcriptomic phenotype at single-cell resolution, and gives an indication of T cell frequency (**Fig. 1a**). To interrogate TCR specificity in a highly multiplexed fashion, we generated libraries of HLA-conjugated streptavidin tetramers (**Fig. S1**) using ultraviolet-mediated peptide exchange with subsequent labeling via biotinylated DNA barcodes. The encoded, peptide-exchanged tetramers showed similar staining to conventional tetramers (**Fig. S2**) while enabling readout via single-cell sequencing.

We designed peptide-HLA tetramer libraries to ensure comprehensive coverage of SARS-CoV-2 and related human coronaviruses across four class I HLA alleles prevalent in North America (A*02:01, B*07:02, A*01:01, and A*24:02, hereafter A*02, B*07, A*01, and A*24). Library inclusion was determined computationally using predicted HLA binding (NetMHC-4.0 (*32*)) of candidate peptides from a set of all possible 9-mers from the SARS-CoV-2 proteome (40% from structural, 60% from non-structural proteins), potentially immunogenic neopeptides from known SARS-CoV-2 variants, and immunogenic epitopes from SARS-CoV-1. A total of 1,355 SARS-CoV-2-related epitopes were included in the libraries in addition to well-characterized epitopes from common endemic viruses (cytomegalovirus (CMV), Epstein-Barr virus (EBV), and influenza).

The peptide-HLA tetramer libraries were used to interrogate peripheral blood mononuclear cells (PBMCs) from individuals who had been infected with SARS-CoV-2 (n = 28 convalescent, n = 27 with acute disease that required hospitalization), or who were unexposed (n = 23) (summarized in **Data file S2**). For each sample, CD8^+^ cells were isolated from PBMCs using magnetic separation, incubated with HLA-matched tetramer libraries, and sorted by flow cytometry to enrich viable, tetramer positive cells (**Fig. S3**). Sorted single cells were encapsulated with DNA-encoded hydrogel beads to provide cell-specific barcodes and unique molecular identifiers (UMIs) that could be used to unify reads across independent sequencing libraries for TCR, peptide-HLA tetramer, and messenger RNA (mRNA) (**Fig. 1a**). We determined the specificity of TCRs using a classification method that identified UMI counts for TCR-peptide-HLA interactions that were outliers when Z-score transformed within and across cells for each sample. The resulting classifier was evaluated against functional assay data for each allele by a receiver-operator curve (ROC) analysis to identify thresholds, which were then used for normalization. The normalized classifier evaluated by ROC analysis provided an area under the curve (AUC) of 0.82 (**Fig. S4)**, and at a threshold of 1, yielded a true positive rate of 93% and a false positive rate of 32%.

From the 52,728,647 CD8^+^ cells interrogated from acute and convalescent COVID-19 patients, we identified high-confidence TCR-peptide-HLA interactions across 434 immunogenic SARS-CoV-2-derived epitopes and 1,163 independent α/β TCR clonotypes (**Fig. 1b****, Data file S3**). The immunodominant epitopes we identified ex vivo were consistent with those measured by other means (*8*–*20*), but we also identified many epitopes with less dominant representation (yet observed with two or more reactive clonotypes), 178 of which had not been previously reported as minimal epitopes (**Data file S4**). Importantly, CD8^+^ T cell reactivity to SARS-CoV-2 epitopes was observed across the entire proteome, generally distributed in a manner consistent with protein lengths (**Table S1**). Of relevance, 85 of these epitopes were derived from the Spike protein currently used in vaccines, but only five of them (a total of 35 CD8^+^ T cell clonotypes in our study) would be affected by the delta (B.1.617.2) SARS-CoV-2 variant (**Data file S5**). Interestingly, the epitope NYNYLYRLF in A*24 (NYN-A24) is one of those epitopes, which was detected in 71% of convalescent patients with A*24, but at a modest mean frequency of 0.025%.

Dimensionality reduced projections of mRNA expression for 224,780 CD8^+^ T cells revealed the broad phenotypic variance observed within this study spread across 8 clusters (**Fig. 1c**). We defined the phenotypic features of clusters using gene signatures generally associated with various CD8^+^ T cell states, including those with naïve, memory, effector, and proliferative status (**Fig. 1c**). In this space, cells from convalescent patients that recognized different dominant epitopes were commonly associated with divergent phenotypes, as shown for representative epitopes (**Fig. 1c**). For example, T cells specific for QYIKWPWYI in A*24 (QYI-A24, Spike) were clustered in regions with high effector scores while those specific for PTDNYITTY in A*01 (PTD-A01, Orf1ab) and LLYDANYFL in A*02 (LLY-A02, Orf3a) resided at opposite ends of memory-rich regions. Thus, and as will be further detailed below, the different immunoreactive epitopes of SARS-CoV-2 elicit distinct CD8^+^ T cell phenotypes.

### Evolution of immunoreactivity through COVID-19 disease progression

**Fig. 2.**
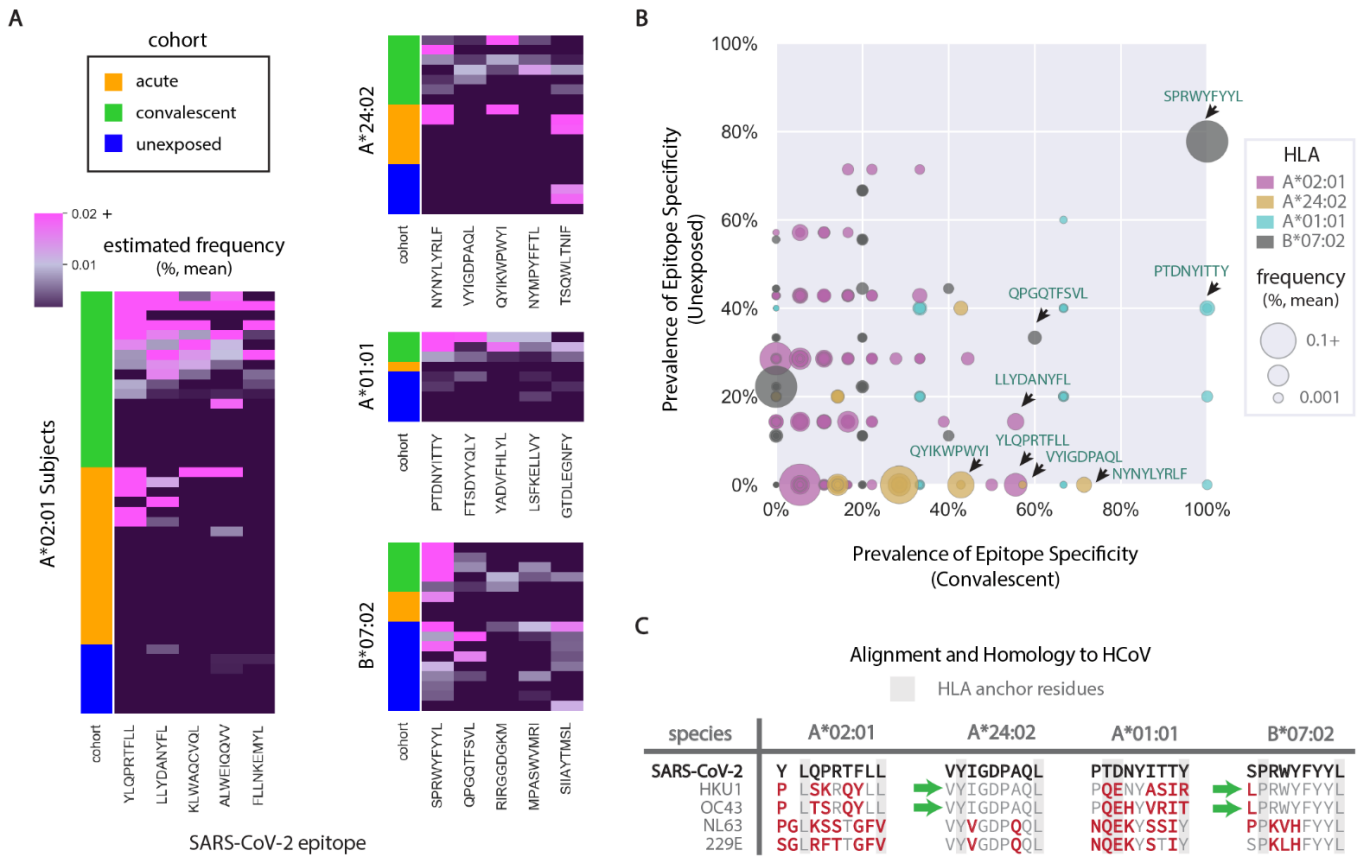
CD8^+^ T cell specificity for major SARS-CoV-2 epitopes across HLA, cohort, and subject. (A) Estimated frequency of T cell response in parent CD8^+^ T cell samples by subject and cohort. Heat maps show mean frequency of epitope specificity calculated for the top five epitopes (x-axis, ranked by cumulative frequency in convalescent patients) by subject (y-axis) across each cohort. Sample counts for convalescent, acute, and unexposed cohorts: A*02:01 (n=18,18,7), A*24:02 (n=7,6,5), A*01:01 (n=3,1,5), B*07:02 (n=5,3,9). (B) Prevalence of T cell specificity observed in unexposed versus convalescent cohorts represented as percentage of cohort with any detectable frequency of T cell specificity against each epitope. Dot sizes represent the mean combined estimated frequency of an epitope specificity across convalescent and unexposed subjects. Sequences of select epitopes from each HLA are annotated. (C) Sequence alignment between select SARS-CoV-2 epitopes and related common cold coronaviruses (HCoV) epitopes. Mismatches are represented in red and HLA anchor residues with a grey background. Green arrows indicate sequences where anchor and all internal residues are conserved between SARS-CoV-2 and HCoV species.

Having established a broad landscape of SARS-CoV-2-reactive CD8^+^ T cells, we asked how TCR repertoires evolve over the course of infection and recovery. As our approach does not require cell expansion to determine TCR specificity, we were able to estimate the frequency of epitope-specific cells in the CD8^+^ T cell pools of convalescent, acute, and unexposed individuals (see **Methods**). While these calculated frequencies are not precise measurements, they are indicative of T cell frequency in parental samples, reflecting robustness of response observed by subject, HLA, and epitope. We show the frequency, for each subject, of T cells reactive to the top five epitopes detected across each of the four HLA variants analyzed (**Fig. 2a**). Notably, we detected markedly fewer SARS-CoV-2-specific T cells in patients with acute disease compared to those in convalescence, which may have been impacted by the lower recovery of T cells that could subsequently be profiled (**Fig. S5**). The striking reduction also applied to memory T cells from prior antiviral responses in these patients, including influenza and EBV, but potentially less to the CMV-specific pool in multiple acute subjects (**Fig. S6**). The paucity of virus-reactive T cells is consistent with the T cell lymphopenia that has been reported to occur in patients with acute COVID-19 (*1*, *33*).

We also observed that the frequencies of SARS-CoV-2-specific T cells in unexposed individuals varied markedly with the HLA allele (**Fig. 2a**). While the top five dominant epitopes in HLA-A*02, A*24, and A*01 were generally associated with measurable responses across convalescent subjects (**Fig. 2b**), the frequency of responses was significantly lower in unexposed compared to convalescent individuals (p=1.5e-5, 8.5e-4, 1.7e-6 by Wilcoxon rank-sum, respectively). In stark contrast, there was no discernible difference in response frequency detected across the most immunodominant epitopes in B*07:02 individuals (p=0.23). In fact, CD8^+^ T cells recognizing nucleocapsid-derived SPRWYFYYL in B*07 (SPR-B07) were found in almost 80% of unexposed subjects (n=9 samples) with a mean frequency of 0.01% (**Fig. 2b**), presaging the immunodominance of this epitope in convalescent COVID-19 patients, where reactivity was detected in 100% of the samples (n=5) at a mean frequency of 0.29%.

The broad presence of SARS-CoV-2-specific T cells in unexposed B*07 subjects could originate from fortuitous cross-reactivity of a public specificity, or from priming via previous exposure to a highly related endemic HCoV. Indeed, SPR-B07 shows marked homology to the corresponding segments of the nucleocapsid proteins from multiple prevalent HCoVs, including HKU1 and OC43, with only a single amino acid residue mismatched at the N terminus (**Fig. 2c**). The nature of the homology preserves internal TCR-contact residues as well as the P and L anchors for HLA binding in peptide positions 2 and 9. Accordingly, the HCoV epitope (LPRWYFYYL, LPR-B07) is predicted to bind with high affinity to HLA-B*07 and could reasonably be expected to cross-react with SPR-B07-specific TCRs. Broader sequence alignment with HCoVs revealed very little homology to the immunodominant epitopes of A*02 and A*01 but did identify a complete match to VYIGDPAQL for A*24 (VYI-A24, Orf1ab). Surprisingly, T cell specificity to VYI-A24 was not detected in a single unexposed subject (n=5 samples). This likely reflects the lower frequency of response elicited by this epitope or an insufficient commitment to memory following exposure to HCoVs. Overall, we found that the response to SARS-CoV-2 is sharply distinguished by HLA genotype, as can be seen clearly in the case of A*02 and B*07, where it appears that highly specific CD8^+^ responses are either generated de novo or amplified from an abundant pre-existing pool, respectively.

### Functional reactivity and cross-reactivity of SARS-CoV2-specific clonotypes

**Fig. 3.**
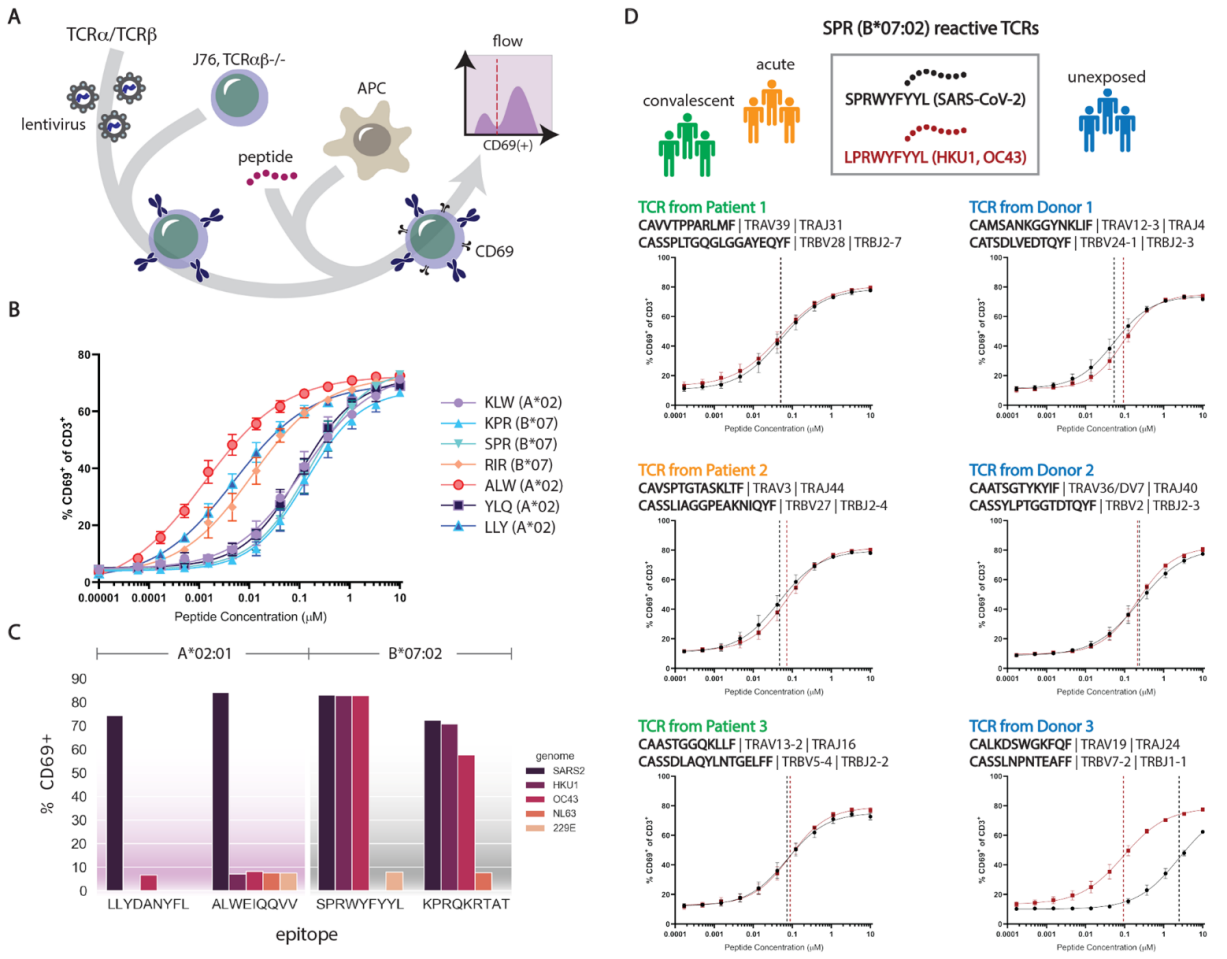
Functional confirmation of identified epitope/HLA interactions with clonotypic TCRs and comparison of SARS-CoV-2 epitopes with common cold coronaviruses. (A) Schematic showing lentiviral transduction of clonotypic recombinant TCRs (rTCRs) into J76 cells, loading of antigen presenting cells (APC) with synthetic peptide, and quantification of activated J76 cells expressing surface CD69. (B) Dose-response curves for TCR-pHLA interactions observed across several canonical epitopes in A*02:01 and B*07:02 backgrounds. Shown are fractions of CD69^+^ cells after a 16-hour stimulation. Error bars represent standard deviation across replicate measurements. (C) Functional activation of TCRs by canonical and homologous epitopes, represented as fraction of CD69^+^ cells after 16-hour stimulation with 10 μM peptide. (D) Dose-response curves for several rTCRs from COVID-19 patients (left) or unexposed subjects (right) stimulated with peptides from SARS-CoV-2 or HCoV HKU1/OC43. Error bars represent standard deviation across replicate measurements.

To confirm the specificity and functionality of TCR-peptide-HLA interactions identified in this study, we cloned several of the observed α/β TCRs clonotypes and expressed them in the TCR-null Jurkat J76 cell line (*34*). Activation of these transductants upon stimulation by SARS-CoV-2 peptides, presented by an HLA-matched lymphoblast cell line, was evaluated by measuring the induction of surface CD69 (**Fig. 3a**). Altogether, we validated 28 interactions for epitopes derived from Orf1ab, Spike, Nucleocapsid, Membrane, and Orf3a proteins, spanning high confidence interactions observed across multiple cells as well as interactions observed exclusively in single cells (**Data file S6**). Dose-response curves for a subset of interactions in A*02 and B*07 are shown in **Fig. 3b**. The effective concentrations (EC50s) measured for these interactions ranged from 1 to 100 nM, with no particular relationship to epitope immunodominance or clonotype frequency measured ex vivo from the respective subject. These values are consistent with interactions measured for CMV-specific epitopes in A*02 using the same system. We next used these recombinant TCR expressing cell lines to compare the functional reactivity elicited by homologous epitopes from HCoVs (**Fig. 3c**). Activation was insignificant for the closest homologs of Orf3a-derived LLY-A02 and Orf1ab-derived ALWEIQQVV in A*02 (ALW-A02, Orf1ab), all of which actually originated from HCoV spike proteins. In contrast, HKU1 and OC43 homologs of nucleocapsid-derived SPR-B07 and KPRQKRTAT in B*07 (KPR-B07, Nucleocapsid) epitopes drove substantial T cell activation (**Fig. 3c**).

We further assessed the sensitivity of B*07 interactions, comparing the reactivity of SPR-B07-specific clonotypes identified from COVID-19 patients or unexposed subjects to SARS-CoV-2-derived SPR-B07 or HCoV-derived LPR-B07 (**Fig. 3d**). The three TCRs identified from COVID-19 individuals yielded EC50s that were essentially identical for the two epitopes, all falling between 50-100 nM (**Fig. 3d****, left**). Two of the TCRs from unexposed individuals yielded EC50s in the same range, again comparable for the HCoV and SARS-CoV-2 variants, while a third showed a >10-fold preference for the HCoV epitope (even though it was originally detected as binding to the SARS-CoV-2 peptide). Aside from providing validation that the specificities detected in our barcoded tetramer technology indeed correspond to antigen-reactive T cells, these findings support that the homologies between SARS-CoV-2 and HCoV epitopes are functionally relevant, and that pre-existing cellular reactivity to SARS-CoV-2 in B*07 subjects likely result from previous exposure to HCoVs like HKU1 or OC43.

### HLA Restricted SARS-CoV-2 Epitopes Impact V(D)J Gene Usage

**Fig. 4.**
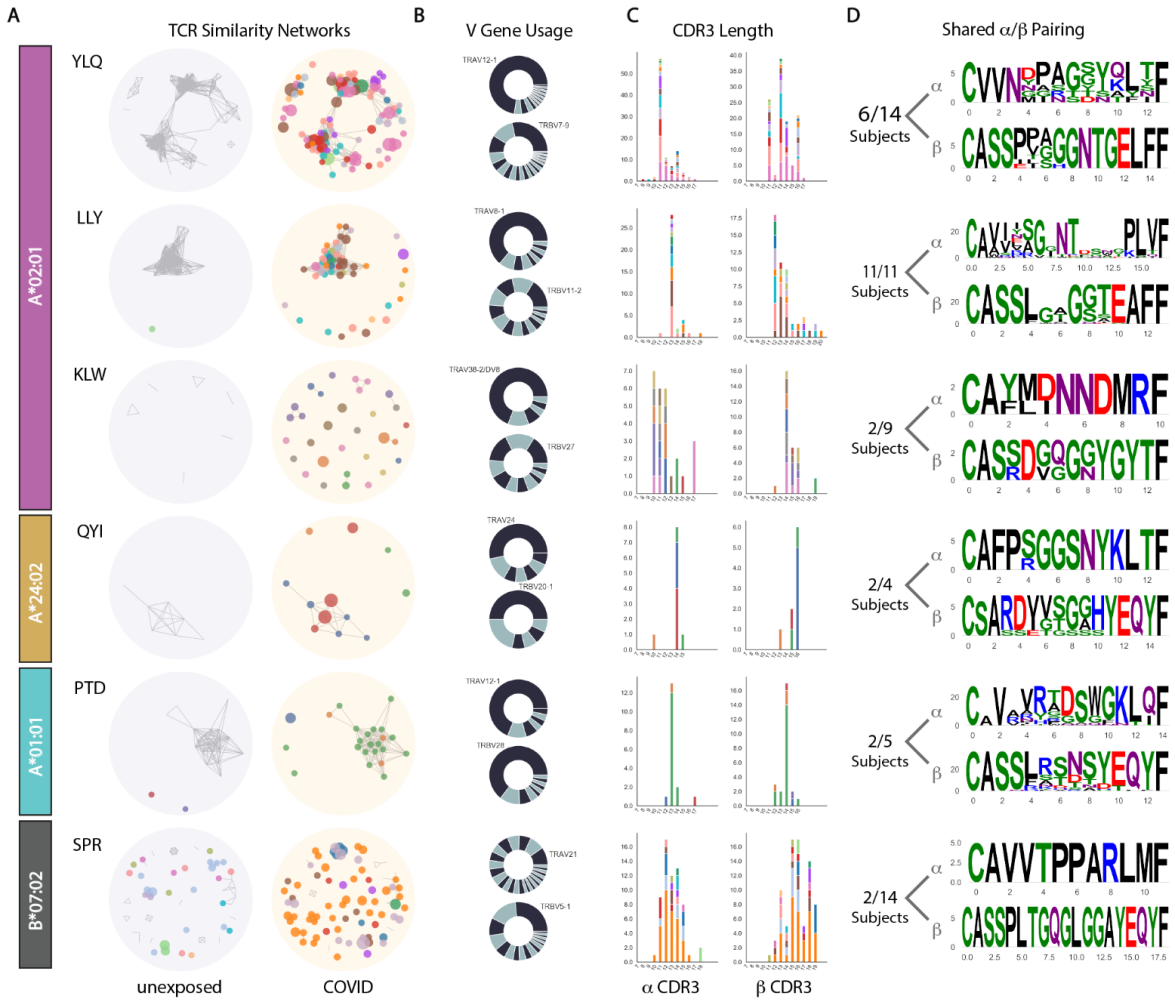
Analysis of TCR sequences from cells specific for the most immunodominant epitopes for each HLA allele tested. (A) Network plots showing biochemical similarity of TCRα or TCRβ CDR3 regions in unexposed subjects (left) or COVID-19 patients (right). Unique subjects are identified by node color. Each node is a unique clonotype within a subject, and the size of the node represents the relative frequency of the response detected. Edges drawn between nodes represent CDR3 homology, and the size of each node represents relative cell frequency. (B) TCRα or TCRβ (V) gene usage across all sequences represented in (A) with the most frequently used gene labeled. (C) Distributions of CDR3 lengths. (D) Paired CDR3 motifs for the most interconnected nodes identified in the network analysis (A).

Given the comprehensive landscape of TCR specificity determined with our approach, we sought to elucidate the extent to which TCR usage is shared within and across subjects. We examined the linkage between paired TCR α/β sequences and their epitope specificity to determine if any features are implicated in the CD8^+^ T cell response to SARS-CoV-2. We used TCRs from 2,469 SARS-CoV-2-specific T cells to perform network mapping of epitope-specific subsets across several immunodominant epitopes identified (**Fig. 4a**). Importantly, because it is known that during development, a TCR β-chain can be paired with many different α-chains, the network analysis allowed clonotype linkages by α or β complementarity-determining region 3 (CDR3) sequences (indicated by edges), identifying conserved motifs based on physicochemical similarity (via BLOSUM matrices) within in the epitope specific T cell population (*35*). T cells from COVID-19 patients that recognize the most dominant A*02-, A*24-, and A*01-restricted epitopes, which have no counterpart in unexposed repertoires, showed a high degree of motif sharing with the exception of KLWAQCVQL in A*02 (KLW-A02, Orf1ab) (**Fig. 4a**). Interestingly, all of these epitopes, including KLW-A02, show dominant usage of a single TCR alpha variable (TRAV) region, and in the cases of QYI-A24 and PTD-A01, dominant usage of both TRAV and TCR beta variable (TRBV) regions (**Fig. 4b****)**. In marked contrast, SPR-B07-specific T cells, including those that also recognize homologs from HCoV, were far more diverse in CDR3 across subjects (**Fig. 4a**), using 8 TRAV and 3 TRBV regions to cover 50% of the clonotypes represented. We observed two instances of CDR3 homology shared across cohorts, as indicated by the presence of nodes with unconnected edges, which are represented in both network maps.

These comparisons show that the reactivities that appear during SARS-CoV-2 infection may stem from both the amplification of highly related TCRs, or from the usage of diverse pre-existing T cell populations. This conclusion extended to CDR3 lengths (**Fig. 4c**), which were tightly distributed for α− and/or β- chains in T cells reactive to the top epitopes in A*02, A*24, and A*01, but substantially less so for SPR-B07. To further elucidate the extent of the public nature of paired α/β TCR usage in COVID-19, we generated consensus sequences from select interconnected network clusters (**Fig. 4d**). This representation provides insight into α/β linkage in the context of public responses that cannot be afforded by bulk sequencing approaches. Most motifs were represented by multiple sequences and shared by at least 40% of the subjects studied, with the exception of KLW-A02 that was shared across only 22%, and SPR-B07 that was shared across only 14%, notably with identical α/β sequences (**Fig. 4d**). Thus, we have observed divergent TCR repertoire utilization, conditioned by HLA and the presence of diverse, pre-existing reactivity resulting from prior viral exposure.

### CD8^+^ Memory T cell Phenotypes vary with recognition of SARS-CoV-2 epitopes

**Fig. 5.**
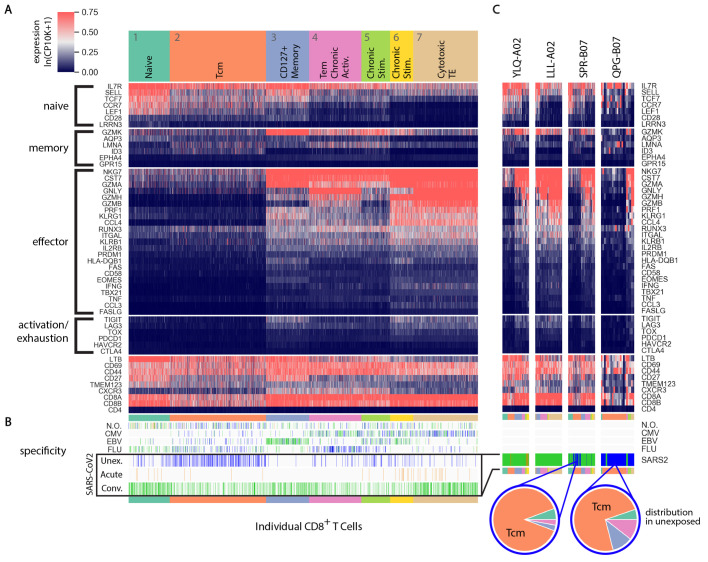
Transcriptomic clustering of T cells based on function-specific gene sets. (A) Single cell gene expression heat map of single CD8^+^ T cells specific for SARS-CoV-2, CMV, EBV, Influenza, or with no observed (N.O.) specificity. Units are log transformed, normalized counts per 10,000 UMIs per cell plus one, ln(CP10K+1). Kmeans clustering identified seven distinct clusters showing gene expression consistent with a range of functional states. (B) Ticks indicate the location and cohort assignment of individual cells with specificity for epitopes from SARS-CoV-2, CMV, EBV, influenza or no observed (N.O.) specificity. (C) Gene expression of single cells with individual epitope specificities indicated in the heat map. In cases where specificity was detected in the unexposed cohort, pie charts are shown to indicate the fraction of cells corresponding to each cluster identified in (A).

To examine how CD8^+^ T cell phenotype varied in relation to disease status, HLA/epitope specificity, and TCR diversity, we performed a more detailed analysis of the single-cell transcriptomic data. We leveraged, as an internal reference, the transcriptomic phenotype of T cells reactive to common acute and latent infections, including influenza, EBV, and CMV. To relate these data to existing knowledge on differentially expressed genes that delineate CD8^+^ T subsets, we used supervised partition clustering based on imputed expression (see **Methods**) of a set of 51 curated transcripts (**Table S2**) characteristic of naïve, memory, effector, or chronically-activated/exhausted populations (**Fig. 5a**). This resulted in the identification of seven distinct cell clusters. Some were easily assigned (naïve cells in C1, central memory in C2, and fully activated cytotoxic effectors in C7). Other memory/effector intermediates were more tentatively labeled, as they did not easily fit into existing categorizations (*36*–*38*). These included a population (C3, here “CD127^+^ Memory”), which expresses markers of naïve, memory and effector cells, and 3 other clusters with characteristics of memory or chronically activated cells (C4-6).

SARS-CoV-2 specific T cells were found in all clusters (**Fig. 5b**, bottom), but at proportions that varied with stage of disease and epitope specificity (**Data file S7**). Cells from acute patients predominantly showed effector phenotypes, but also paradoxically naïve types. In convalescent donors, T cells from several epitope specificities were broadly distributed, consistent with the resolution of an infection. Several epitope-specific T cell pools were predominantly found in central memory (C2), including PTD-A01 (49%) and LLY-A02 (42%), while others predominantly resided in the cytotoxic terminal effector cluster (C7), including TLMNVITLV in A*02 (TLM-A02, Orf1ab, 80%), and LLLDRLNQL in A*02 (LLL-A02, Nucleocapsid, 61%) (**Fig. 5c**). In most other reactivities, including SPR-B07, transcriptional profiles in convalescent patients were fairly broadly distributed across all clusters. In contrast, the reactivity in unexposed subjects was dominated by the central memory pool, confirming that the CD8^+^ cells likely result from long-term exposure to cross-reactive antigens. This was especially clear in the case of B*07, where epitope-specific T cells for SPR-B07, QAWQPGVAM in B*07 (QPG-B07, Orf1ab), and SIIAYTMSL in B*07 (SII-B07, Spike) were represented in central memory (C2) at proportions of 88%, 75%, and 67%, respectively. Other notable reactivities associated with central memory include TSQWLTNIF in A*24 (TSQ-A24, Orf1ab, 70%) and NSSTCMMCY in A*01 (NSS-A01, Orf1ab, 68%), though the source of these memory cells, like QPG-B07 and SII-B07, does not appear to be from HCoV exposure based on a lack of homology. Overall, this analysis provides further evidence that SPR-B07 responses to SARS-CoV-2 are likely drawn from a cross-reactive memory pool and that commitment to different cell fate is dependent on epitope specificity.

We also observed an interesting dynamic between SARS-CoV-2 infection and existing T cell pools specific for common viral infections, with differentiated outcomes likely shaped by exposure history (**Fig. 5b**). Influenza-specific CD8^+^ T cells, which result from vaccination or past infections, mapped primarily to the central memory (C1) and effector memory (C3) compartments in unexposed individuals. Proportions were stable across epitope specificities in COVID-19 patients with the exception of GILGFVFTL in A*02 (GIL-A02, Influenza A Matrix protein 1), where the proportion of effector memory cells decreased from 50% to 0% and a naïve population representing 30% of the cells paradoxically emerged. CMV- and EBV-specific T cells, likely subject to more chronic stimulation from low-level re-activation of these integrated herpesviruses, mapped to more activated pools in unexposed subjects, as has been described by others (*39*). After SARS-CoV-2 infection, EBV-specific cells shifted markedly from central memory (C2) and chronically stimulated compartments (C5) into the CD127^+^ memory cluster (C3). These changes may reflect either bystander activation, perhaps as a result of the high cytokine release in COVID-19 patients, or from changes in homing or recirculation patterns that bring into the blood cells normally sequestered in tissues. These observations suggest that, in addition to inducing lymphopenia, COVID-19 strongly reshuffles third-party antiviral T cell pools, the extent of which may be associated with exposure history and, at least to some degree, epitope specificity.

## DISCUSSION

Here we presented a unified description of the CD8^+^ T cell response to SARS-CoV-2, highlighting the importance of HLA genetics, TCR repertoire diversity, and epitope-specific navigation through a complex transcriptomic phenotype at various stages of disease. In building a comprehensive map of immunodominant, HLA-restricted epitopes broadly derived from proteins across the entire SARS-CoV-2 proteome, we highlight how only some HLA haplotypes are associated with the existence of a pre-existing CD8^+^ T cell memory pool in unexposed individuals. We further show how HLA variation plays an important role in shaping the diversity of CD8^+^ T cell repertoires upon exposure to SARS-CoV-2, and that cellular phenotype and commitment to memory can be associated with epitope-specificity in the context of both SARS-CoV-2 and latent EBV infections.

The presence of SARS-CoV-2 reactive CD8^+^ T cells has been linked to milder disease (*5*, *11*, *12*), although the precise link between cellular immunity and host protection still remains to be further understood (*7*, *40*, *41*). We found that individuals carrying HLA-B*07 show a CD8^+^ T cell response that is dominated by pre-existing memory pools reactive to multiple SARS-CoV-2 epitopes, especially SPR-B07, which is likely induced by previous exposures to benign HCoVs. In contrast, the immunodominant responses in A*02 individuals (e.g., to YLQPRTFLL in A*02 (YLQ-A02, Spike) and LLY-A02) appear to be driven largely by the expansion of antigen-inexperienced SARS-CoV-2-specific T cells. It is interesting to note that CD8^+^ T cell cross-reactivity may be less widespread in unexposed individuals than for CD4^+^ T cross-reactivity, for which ~50% of unexposed individuals exhibited CD4^+^ T cell memory (*16*). Our data provides a basis for this limited representation of the CD8^+^ T cell repertoire in that only a subpopulation of individuals carrying a specific HLA allele would have these cross-reactive memory CD8^+^ T cells. The extent to which pre-existing memory specific to SPR-B07 contributes to protection would need to be explored with longitudinal studies spanning SARS-CoV-2 exposure.

The interplay between HLA-restricted epitope presentation and available TCR repertoire shapes the cellular response to SARS-CoV-2. There are few limited studies suggesting an influence of HLA genotype on COVID-19 severity (*28*, *42*–*44*). Large-scale studies evaluating T cell responses across a comprehensive HLA coverage per patient may help identify or deconvolute relationships between HLA genotype, like B*07 in this study, and protection against severe disease, ideally uncovering mechanism. Here, we observed an interesting connection between TCR repertoire diversity and HLA restriction. Responses seen in A*02, A*24, and A*01 were more often associated with “public” CDR3 motifs and consistent V gene segment usage in the α− and/or β− chains. In contrast, the dominant immune response in B*07 leveraged a significantly more diverse TCR repertoire. Several contributors to public TCR responses have been proposed, focusing on the physicochemical features of HLA-restricted peptides (e.g., “featureless” peptide-HLAs may drive a public response) and convergent recombination of TCR sequences (*45*). The method described in this work provides an ideal system to address this question. Perhaps counterintuitively, our results show that in the case of COVID-19, the largest pool of potentially protective, pre-existing cellular immunity is derived from one of the *least* public epitope-specific repertoires, possibly reflecting the influence of repeated acute infections with HCoVs throughout the life of the individuals.

Beyond the comprehensive deciphering of TCR specificity reported here, we also provided a detailed picture of the complex and dynamic transcriptional landscape of the CD8^+^ T response to SARS-CoV-2. Importantly, we were able to demonstrate that the pre-existing SPR-B07 reactivity, observed in ~80% of unexposed subjects with HLA-B*07, was predominantly associated with a central memory-like transcriptional profile (88% of SPR-B07-reactive T cells), confirming that it originates from prior exposures. In convalescent patients, we observed a much broader distribution of SPR-B07-reactive T cells spanning every functional state at proportions ranging from 5-29% (**Data file S7**). This is consistent with late contraction/early memory formation described for SARS-CoV-2 in a recent study (*12*), where cells spanned naïve, central memory, various classifications of effector memory, and terminally differentiated effector memory expressing RA (TEMRA). There was no evidence for a particularly frequent “exhausted” state among SARS-CoV-2-specific CD8^+^ T cells, as suggested elsewhere (*46*, *47*) (acknowledging that the phenotypic state is a proxy for true reactivity testing, and that blood T cells may not fully reflect what happens in the lung). We also did not find evidence of “antigenic sin” resulting from HCoV pre-exposure (*48*) that would stifle an effective response to SARS-CoV-2-unexposed B*07 individuals. Whether HLA haplotype plays a role in the durability of the CD8^+^ T cell responses, especially to SARS-CoV-2 vaccines, may have impact for long-term protection across different ethnic groups and geographic regions.

Another interesting observation from this work, as noted by others (*49*), is that even at the height of infection or shortly after viral clearance, the cumulative anti-SARS-CoV-2 CD8^+^ T cell response barely reached the frequency of anti-influenza memory responses and was well below the frequencies that could be achieved by CMV-specific cells in the same individuals (**Fig. S6**). This was notably evident in the acutely infected individuals, at a time where the contribution of cytotoxic CD8^+^ T cells would have been most important. We acknowledge the caveat that peripheral frequencies were measured, and some degree of sequestration in viral target tissues, such as the lung, is likely to occur in acute patients. Yet, the response seems much more muted than the robust response observed in some other viral infections (*50*). This meager outcome was seen both for the cross-reactive “secondary responses” by memory T cells pre-primed by endemic HCoVs, as well as for the primary responses of truly SARS-CoV-2 species-specific CD8^+^ T cells amplified de novo. This suggests that the paucity likely does not result from a blocking of primary activation, but from a dampening of all specific CD8^+^ T cells. Consistent with this notion, the detection of influenza/EBV/CMV reactive cells were also lower in acute COVID-19 patients, compared to SARS-CoV-2 “naïve” individuals. It has been proposed that the lethal cytokine storm in severe COVID-19 stems from innate immune functions overcompensating for adaptive immune system failures (*2*).

Given the widespread lymphopenia observed in acute COVID-19, we considered the possibility of latent virus reactivation with the loss of protective CMV- and EBV-specific T memory pools. While we have no direct evidence of impact on disease outcome, we do observe a significant alteration of cell state within these subsets. While CMV-reactive cells remained within, though somewhat shuffled, the same effector/memory transcriptional phenotypes between unexposed and COVID-19 cohorts (including chronic stimulation, cytotoxic terminal effector, and terminal effector memory), we observed a striking shift of EBV-specific cells from chronic stimulation and central memory into the “CD127^+^ memory” state in COVID-19-exposed individuals. These cells expressed moderate to high levels of many naïve (IL7R, SELL, CCR7), memory (GZMK), and effector-associated genes (NKG7, CST7, GZMA), along with markers of activation/exhaustion (TIGIT, LAG3), making them particularly interesting and difficult to ascribe to conventional phenotype labels. Recently, two transcriptionally distinct stem-like CD8^+^ T cell memory states were described, one of which was functionally committed to a dysfunctional lineage (*38*). As these cell states were differentiated by many of the same markers observed in our “CD127^+^ memory” compartment, it would be interesting to determine to what extent these “CD127^+^ memory” cells, dominated by EBV-reactive pools, experience similar fates of dysfunction. We speculate that this phenotype may be a consequence of the particular inflammatory milieu of COVID-19 patients.

There are several limitations to this study. While we have investigated the CD8+ T cell response to epitopes predicted to be presented with high affinity in four common HLA alleles, the selection of HLAs and epitopes was not exhaustive. We assessed predominantly 9-mer epitopes from canonical open reading frames of a single SARS-CoV-2 variant. Subsequent studies may include a more comprehensive set of epitopes, broader coverage of HLAs, the exploration of non-canonical open reading frames, and inclusion of several SARS-CoV-2 variants. Another limitation of this study is the small sample size for specific HLA alleles and limited cell recovery for samples from acute patients. Our findings on response prevalence, public features of T cell repertoires, and T cell phenotype could be further substantiated or broadened with deeper sampling across genetically diverse populations and larger cell inputs. Related to this, there are limitations in the interpretation of response frequencies calculated in this work, especially in the cases of low cell input. The frequencies calculated are intended to provide a qualitative assessment of T cell response, allowing for comparisons across subjects, HLAs, and epitopes.

In conclusion, we leveraged a powerful single-cell technology to better elucidate the roles of HLA variation, TCR diversity, and cellular phenotypes in establishing pre-existing immunity to SARS-CoV-2. We observed the presence of a diverse and immuno-dominant nucleocapsid epitope-specific memory pool in subjects with HLA-B*07 but saw little evidence of similar reactivity in individuals with other HLA alleles. Outside of the HLA-B*07, the epitope-specific TCR repertoires observed were largely public in nature. We measured a diverse landscape of T cell phenotypes associated with SARS-CoV-2 infection, and also observed an influence on T cell repertoires reactive to persistent and latent infections with other viruses. Overall, this work provides a framework for the unified characterization of the cellular response to novel viral infections. The ability to understand the basis of cellular immunity to SARS-CoV-2 and other pathogens will provide insight for the continued assessment of immune surveillance, health security, and long-term protection from future respiratory pathogens.

## MATERIALS AND METHODS

**Study design.** The aim of this study was to identify features of CD8^+^ T cell responses to SARS-CoV-2 associated with disease state and HLA genetics, including immunodominant T cell epitopes, evidence of immune recall, and shared TCR sequence motifs. We used libraries of peptide-HLA tetramers with epitopes derived from across the SARS-CoV-2 proteome presented in four HLAs with high prevalence in North America. Samples from acute and convalescent patients, with HLAs matching the tetramer libraries, were acquired as they became available and screened in several batches alongside samples from unexposed subjects. A total of 27 acute, 28 convalescent, and 23 unexposed subjects were screened providing HLA-matched analysis for 43 A*02:01, 18 A*24:02, 17 B*07:02, and 9 A*01:01 samples.

**Antigen library design.** Antigenic peptide libraries were designed by scoring all possible 9mer peptides derived from the entire SARS-CoV-2 proteome (NC_045512.2) using netMHC-4.0 (*32*) in the HLA-A*02:01, HLA-A*01:01, HLA-A*24:02 or HLA-B*07:02 alleles. SARS-CoV-1 peptides that had evidence of T cell positive assays, obtained from the Immune Epitope Database (www.iedb.org; (*51*)), and that were highly homologous to their SARS-CoV2 counterparts within hamming-distance of 2 were converted to 9-mers. Additionally, SARS-CoV-2 peptides predicted to raise immunogenic responses by others were also included (*52*, *53*). Finally, libraries included a set of well-defined viral epitopes from Cytomegalovirus, Epstein-Barr virus, and Influenza viruses (CEF peptide pool) that elicit T cell responses in the population at large. Antigenic peptides with 500 nM affinity or lower were then selected for inclusion (**Data file S8**).

**Production of tetramer library pools.** HLA-A*01:01, -A*02:01, -A*24:02 and HLA-B*07:02 extracellular domains were expressed in *E. coli* and refolded along with beta-2-microglobulin and ultraviolet (UV)-labile place-holder peptides STAPGJLEY, KILGFVFJV, VYGJVRACL and AARGJTLAM, respectively (*54*). A C-terminal sortase recognition sequence on the HLA was modified by sortase transpeptidation (*55*, *56*) with a synthetic alkynylated linker peptide, featuring an N-terminal triglycine connected to propargylglycine via a PEG linker (Genscript, Piscataway, NJ). The modified HLA monomer was then purified by size exclusion chromatography (SEC). Full-length streptavidin with an N-terminal Flag tag and a C-terminal sortase recognition sequence and 6xHisTag was prepared by expression and purification from *E. coli* using immobilized metal affinity chromatography and SEC. Streptavidin was modified by sortase transpeptidation with a synthetic azidylated linker peptide, featuring an N-terminal triglycine connected to picolyl azide via a PEG linker (Click Chemistry Tools, Scottsdale, AZ). HLA tetramers were produced by mixing alkynylated HLA monomers and azidylated streptavidin in 0.5 mM copper sulfate, 2.5 mM BTTAA (2-(4-((Bis((1-(tert-butyl)-1H-1,2,3-triazol-4-yl)methyl)amino)methyl)-1H-1,2,3-triazol-1-yl)acetic acid) and 5 mM ascorbic acid for up to 4 hours on ice, followed by purification of highly multimeric fractions by SEC. Individual peptide exchange reactions containing 500 nM HLA tetramer and 60 uM peptide were exposed to long-wave UV (366 nm) at a distance of 2-5 cm for 30 min at 4°C, followed by 30 min incubation at 30°C. A biotinylated oligonucleotide barcode (Integrated DNA Technologies) was added to each individual reaction followed by 30 min incubation at 4°C. Individual tetramer reactions were then pooled and concentrated using 30 kDa molecular weight cut-off centrifugal filter units (Amicon). Tetramer production was quality controlled using SEC (**Fig. S1a**), sodium dodecyl sulfate polyacrylamide gel electrophoresis (SDS-PAGE) (**Fig. S1b**), and UV-mediated peptide exchange by assessing binding to peptide-expanded cell lines (**Fig. S2**).

**Patient Samples.** Peripheral blood mononuclear cells (PBMCs) from COVID-19 positive donors or unexposed donors were obtained from Precision 4 Medicine (USA), the Massachusetts Consortium on Pathogen Readiness (MassCPR, Boston, USA), or CTL (USA), all under appropriate informed consent. Patients were defined COVID-19 positive based on positive SARS-CoV-2 real-time reverse transcriptase–polymerase-chain-reaction (RT-PCR) using nasopharyngeal swabs. Patient samples were characterized as “acute” if collected while the patient was hospitalized and as “convalescent” if collected after recovery or when presenting mild disease. Samples from unexposed subjects were collected prior to December, 2019. A summary of patient samples used in this study are presented in **Data file S2**.

**Cell Staining.** PBMCs were thawed, and CD8+ T cells were enriched by magnetic-activated cell sorting (MACS) using a CD8+ T Cell Isolation Kit (Miltenyi) following the manufacturers protocol. The CD8+ T cells were then stained with tetramer libraries (**Data file S8**), matched to subject HLAs (**Data file S2**) and at 1nM final concentration for each member, in the presence of 2 mg/mL *salmon sperm* DNA in PBS with 0.5% BSA solution for 20 min. Cells were then labeled with anti-TCR antibody-derived tag (ADT, clone IP26, Biolegend, CA, USA) for 15 min followed by washing. Tetramer bound cells were then labeled with phycoerythrin (PE) conjugated anti-DKDDDDK-Flag antibody (BioLegend, CA, USA) followed by dead cell discrimination using 7-amino-actinomycin D (7-AAD). The live, tetramer positive cells were sorted (**Fig. S3**) using a Sony MA900 Sorter (Sony). When necessary, sorting gates were set liberally to enable sufficient cell recovery for single-cell sequencing.

**Sample multiplexing.** To ensure sufficient cell loading and subsequent cDNA production in single-cell sequencing, we used sample multiplexing for several experiments. When applied, samples were independently stained with tetramer libraries, labeled using custom anti-TCR ADTs with unique 15 base pair DNA barcodes (clone IP26, BioLegend, CA, USA), and sorted. ADT-labeled, sorted samples were combined prior to encapsulation and single-cell sequencing. In several cases, an expanded T cell line (Cellero Anti-MART-1, MA, USA) was labeled with a BV785 anti-CD8 antibody (BioLegend, CA, USA), stained using a tetramer for ELAGIGILTV in A*02:01, and subsequently mixed and co-sorted alongside samples interrogated for this study. This provided confirmation of tetramer staining, guidance for gating, and verification of the multiplexing strategy (**Fig. S3**). The anti-MART-1 T cells (TCR sequences provided in **Data file S9**) were excluded from any subsequent analyses.

**Single-cell Sequencing.** Tetramer positive cells were counted by Nexcelom Cellometer (Lawrence, MA, USA) using AOPI stain following manufacturer’s recommended conditions. When possible, 15,000 cells were targeted for encapsulation. Single-cell encapsulations were generated utilizing 5′ v1 Gem beads from 10x Genomics (Pleasanton, CA, USA) on a 10x Chromium controller and downstream TCR, Gene Expression, and Surface marker libraries were made following manufacturer recommended conditions. All libraries were quantified on a BioRad CFX 384 (Hercules, CA, USA) using Kapa Biosystems (Wilmington, MA, USA) library quantified kits and pooled at an equimolar ratio. TCRs, Gene Expression, surface markers, and tetramer generated libraries were sequenced on Illumina (San Diego, CA, USA) NextSeq550 instruments. Sequencing data were processed using the Cell Ranger Software Suite (Version 3). Samples were demultiplexed and unique molecular identifier (UMI) counts were quantified for TCRs, tetramers, and gene expression.

**Single-cell Transcriptomic Analysis.** Hydrogel-based RNA-seq data were analyzed using the Cell Ranger package from 10X Genomics (v3.1.0) with the GRCh38 human expression reference (v3.0.0). Except where noted, Scanpy (v1.6.0 (*57*)) was used to perform the subsequent single cell analyses. Any exogenous control cells identified by TCR clonotype were removed before further gene expression processing. Hydrogels that contain UMIs for less than 300 genes were excluded. Genes that were detected in less than 3 cells were also excluded from further analysis. Several additional quality control thresholds were also enforced. To remove data generated from cells likely to be damaged, upper thresholds were set for percent UMIs arising from mitochondrial genes (13%). To exclude data likely arising from multiple cells captured in a single drop, upper thresholds were set for total UMI counts based on individual distributions from each encapsulation (from 1500 to 3000 UMIs). A lower threshold of 10% was set for UMIs arising from ribosomal protein genes. Finally, an upper threshold of 5% of UMIs was set for the MALAT1 gene. Any hydrogel outside of any of the thresholds was omitted from further analysis. A total of 15,683 hydrogels were carried forward. Gene expression data were normalized to counts per 10,000 UMIs per cell (CP10K) followed by log1p transformation: ln(CP10K + 1).

Highly variable genes were identified (1,567) and scaled to have a mean of zero and unit variance. They were then provided to scanorama (v1.7, (*58*)) to perform batch integration and dimension reduction. The data were used to generate the nearest neighbor graph which was in turn used to generate a UMAP representation that was used for Leiden clustering. The hydrogel data (not scaled to mean zero, unit variance, and before extraction of highly variable genes) were labeled with cluster membership and provided to SingleR (v1.4.0, (*59*)) using the following references from Celldex (v1.0.0, (*59*)): Monaco Immune Data, Database Immune Cell Expression Data, and Blueprint Encode Data. SingleR was used to annotate the clusters with their best-fit match from the cell types in the references. Clusters that yielded cell types other than types of the T Cell lineage were removed from consideration and the process was repeated starting from the batch integration step. The best-fit annotations from SingleR after the second round of clustering and the annotation was assigned as putative labels for each Leiden cluster. Further clustering of transcriptomic data was performed across the genes shown in Fig. 5 using KMeans in sklearn (v0.24) with n_clusters set to 8. As the method has a preference to assign like-sized clusters, further consolidation of two central memory clusters was performed.

In order to provide corroboration for the SingleR best-fit annotations and further evidence as to the phenotype of the clusters, gene panels representing functional categories (Naïve, Effector, Memory, Exhaustion, Proliferation) were used to score each hydrogel’s expression profiles using scanpy’s “score_genes” function (*57*) which compares the mean expression values of the target gene set against a larger set of randomly chosen genes that represent background expression levels. The gene panels for each class were: Naïve - TCF7, LEF1, CCR7; Effector - GZMB, PRF1, GNLY; Memory - AQP3, CD69, GZMK; Exhaustion - PDCD1, TIGIT, LAG3; Proliferation - MKI67, TYMS. The gene expression matrix for all hydrogels were first imputed using the MAGIC algorithm (v2.0.4, (*60*). These functional scores were the only data generated from imputed expression values.

**Scoring peptide-HLA-TCR interactions.** Tetramer data analysis was performed using built-in methods of pandas (v1.2.5) and numpy (v1.20.3) in Python (v3.7.3). For each single-cell encapsulation, tetramer UMI counts (columns) were matrixed by cell (rows) and log-transformed. Duplicates of this matrix were independently Z-score transformed by row or column, and subsequently median-centered by the opposite axis (column or row), respectively (**Fig. S7**). For each peptide-HLA-cell interaction, this provided two scores - inter-tetramer ($Z_{tet}$) and inter-cell ($Z_{cell}$), which were used to calculate a classifier for unique CDR3 a/b clonotypes across N cells as $N\times {\bar{Z}}_{tet}\times {\bar{Z}}_{cell}$. Classifier thresholds for positive interactions were set at 40, 36, 50, and 65 for A*02:01, B*07:02, A*24:02, and A*01:01, respectively.

**Frequency Calculation.** The frequency of reactive T cells in parent CD8^+^ T cell populations was estimated using a calculation of compounded frequency by taking the product of the fraction of reactive cells in the sorted population and the fraction of cells sorted (**Fig. S8**). When sample multiplexing was applied, care was taken to include only de-multiplexed cells from the corresponding sample to determine reactive cell fraction.

**TCR Network Analysis.** TCR motif analysis was performed using scirpy (v0.6.1) with receptor_arms = “any,” metric = “alignment,” and default cutoff of 10. Once clusters were identified, sequence alignment was performed using the pairwise2 module in Biopython (v1.78) and visualized using logomaker (v0.8).

**Recombinant TCR validation.** Recombinant TCRs identified from patient samples were ordered from TWIST Biosciences in the pLVX-EF1a lentiviral backbone (Takara) as a bicistronic TCRb-T2A-TCRa vector. Viral supernatants from transfected HEK 293T cells were collected 48 and 72 hours after transfection and added to the parental TCRab^−/−^ Jurkat J76 cell like (*34*) expressing CD8 and a nuclear factor of activated T cells (NFAT)-green fluorescent protein (GFP) reporter, referred to as J76-CD8-NFAT-GFP. Recombinant TCR surface expression was confirmed through flow cytometry by staining transduced J76-CD8-NFAT-GFP cells with anti-CD3-PE (Clone UCHT1) and anti-TCRab-allophycocyanin (APC) antibodies (Clone IP26).

To assess functional activity of recombinant TCRs, J76-CD8-NFAT-GFP expressing recombinant TCRs were incubated at a 1:1 ratio with the HLA-A*02:01^+^and HLA-B*07:02^+^ HCC 1428 BL (ATCC CRL-2327) lymphoblastic cell line, with a final concentration of 0.5% dimethylsulfoxide (DMSO, vehicle) or 50 uM of cognate peptide (New England Peptide, >95% pure). Cell mixtures were incubated in the Sartorius IncuCyte at 37°C, 5% CO_2_ overnight and analyzed for NFAT-GFP expression measured as total integrated intensity (GCU x mm^2^/image) at 12 hours after assay setup. At 16 hours, cells were removed from the IncuCyte and subsequently washed and blocked with staining buffer (BD 554656), stained with anti-CD3-PE-Cy7 (Clone UCHT1) and anti-CD69-APC (Clone FN50) antibodies, and analyzed using the Intellicyt iQue Screener Plus and FlowJo v10. CD69 activity was measured as percent positive of CD3^+^ cells.

## MGH COVID-19 Collection and Processing Team participants

Nikolaus Jilg^1^, Jonathan Li^1^, Alex Rosenthal^1^, Colline Wong^1^, George Daley^2^, David Golan^2^, Howard Heller^2^, Arlene Sharpe^2^, Betelihem A. Abayneh^3^, Patrick Allen^3^, Diane Antille^3^, Katrina Armstrong^3^, Siobhan Boyce^3^, Joan Braley^3^, Karen Branch^3^, Katherine Broderick^3^, Julia Carney^3^, Andrew Chan^3^, Susan Davidson^3^, Michael Dougan^3^, David Drew^3^, Ashley Elliman^3^, Keith Flaherty^3^, Jeanne Flannery^3^, Pamela Forde^3^, Elise Gettings^3^, Amanda Griffin^3^, Sheila Grimmel^3^, Kathleen Grinke^3^, Kathryn Hall^3^, Meg Healy^3^, Deborah Henault^3^, Grace Holland^3^, Chantal Kayitesi^3^, Vlasta LaValle^3^, Yuting Lu^3^, Sarah Luthern^3^, Jordan Marchewka Schneider^3^, Brittani Martino^3^, Roseann McNamara^3^, Christian Nambu^3^, Susan Nelson^3^, Marjorie Noone^3^, Christine Ommerborn^3^, Lois Chris Pacheco^3^, Nicole Phan^3^, Falisha A Porto^3^, Edward Ryan^3^, Kathleen Selleck^3^, Sue Slaughenhaupt^3^, Kimberly Smith Sheppard^3^, Elizabeth Suschana^3^, Vivine Wilson^3^, Mary Carrington^4^, Maureen Martin^4^, Yuko Yuki^4^, Galit Alter^5^, Alejandro Balazs^5^, Julia Bals^5^, Max Barbash^5^, Yannic Bartsch^5^, Julie Boucau^5^, Mary Carrington^5^, Josh Chevalier^5^, Fatema Chowdhury^5^, Edward DeMers^5^, Kevin Einkauf^5^, Jon Fallon^5^, Liz Fedirko^5^, Kelsey Finn^5^, Pilar Garcia-Broncano^5^, Musie S. Ghebremichael^5^, Ciputra Hartana^5^, Chenyang Jiang^5^, Kelly Judge^5^, Paulina Kaplonek^5^, Marshall Karpell^5^, Peggy Lai^5^, Evan C. Lam^5^, Kristina Lefteri^5^, Xiaodong Lian^5^, Mathias Lichterfeld^5^, Daniel Lingwood^5^, Hang Liu^5^, Jinqing Liu^5^, Natasha Ly^5^, Zachary Manickas Hill^5^, Ashlin Michell^5^, Ilan Millstrom^5^, Noah Miranda^5^, Claire O'Callaghan^5^, Matthew Osborn^5^, Shiv Pillai^5^, Yelizaveta Rassadkina^5^, Alexandra Reissis^5^, Francis Ruzicka^5^, Kyra Seiger^5^, Libera Sessa^5^, Christianne Sharr^5^, Sally Shin^5^, Nishant Singh^5^, Weiwei Sun^5^, Xiaoming Sun^5^, Hannah Ticheli^5^, Alicja Trocha-Piechocka^5^, Bruce Walker^5^, Daniel Worrall^5^, Xu G. Yu^5^, Alex Zhu^5^

^1^ Brigham and Women’s Hospital, Boston, MA, USA

^2^ Harvard Medical School, Boston, MA, USA

^3^ Massachusetts General Hospital, Boston, MA, USA

^4^ National Cancer Institute, Frederick, MD, USA

^5^ Ragon Institute of MGH, MIT and Harvard, Cambridge, MA, USA

## References

[1] C. Huang, Y. Wang, X. Li, L. Ren, J. Zhao, Y. Hu, L. Zhang, G. Fan, J. Xu, X. Gu, Z. Cheng, T. Yu, J. Xia, Y. Wei, W. Wu, X. Xie, W. Yin, H. Li, M. Liu, Y. Xiao, H. Gao, L. Guo, J. Xie, G. Wang, R. Jiang, Z. Gao, Q. Jin, J. Wang, B. Cao, Clinical features of patients infected with 2019 novel coronavirus in Wuhan, China. Lancet 395, 497–506 (2020). 10.1016/S0140-6736(20)30183-5 31986264 PMC7159299

[2] A. Sette, S. Crotty, Adaptive immunity to SARS-CoV-2 and COVID-19. Cell 184, 861–880 (2021). 10.1016/j.cell.2021.01.007 33497610 PMC7803150

[3] O. W. Ng, A. Chia, A. T. Tan, R. S. Jadi, H. N. Leong, A. Bertoletti, Y. J. Tan, Memory T cell responses targeting the SARS coronavirus persist up to 11 years post-infection. Vaccine 34, 2008–2014 (2016). 10.1016/j.vaccine.2016.02.063 26954467 PMC7115611

[4] J. Seow, C. Graham, B. Merrick, S. Acors, S. Pickering, K. J. A. Steel, O. Hemmings, A. O’Byrne, N. Kouphou, R. P. Galao, G. Betancor, H. D. Wilson, A. W. Signell, H. Winstone, C. Kerridge, I. Huettner, J. M. Jimenez-Guardeño, M. J. Lista, N. Temperton, L. B. Snell, K. Bisnauthsing, A. Moore, A. Green, L. Martinez, B. Stokes, J. Honey, A. Izquierdo-Barras, G. Arbane, A. Patel, M. K. I. Tan, L. O’Connell, G. O’Hara, E. MacMahon, S. Douthwaite, G. Nebbia, R. Batra, R. Martinez-Nunez, M. Shankar-Hari, J. D. Edgeworth, S. J. D. Neil, M. H. Malim, K. J. Doores, Longitudinal observation and decline of neutralizing antibody responses in the three months following SARS-CoV-2 infection in humans. Nat. Microbiol. 5, 1598–1607 (2020). 10.1038/s41564-020-00813-8 33106674 PMC7610833

[5] C. Rydyznski Moderbacher, S. I. Ramirez, J. M. Dan, A. Grifoni, K. M. Hastie, D. Weiskopf, S. Belanger, R. K. Abbott, C. Kim, J. Choi, Y. Kato, E. G. Crotty, C. Kim, S. A. Rawlings, J. Mateus, L. P. V. Tse, A. Frazier, R. Baric, B. Peters, J. Greenbaum, E. Ollmann Saphire, D. M. Smith, A. Sette, S. Crotty, Antigen-Specific Adaptive Immunity to SARS-CoV-2 in Acute COVID-19 and Associations with Age and Disease Severity. Cell 183, 996–1012.e19 (2020). 10.1016/j.cell.2020.09.038 33010815 PMC7494270

[6] J. M. Dan, J. Mateus, Y. Kato, K. M. Hastie, E. D. Yu, C. E. Faliti, A. Grifoni, S. I. Ramirez, S. Haupt, A. Frazier, C. Nakao, V. Rayaprolu, S. A. Rawlings, B. Peters, F. Krammer, V. Simon, E. O. Saphire, D. M. Smith, D. Weiskopf, A. Sette, S. Crotty, Immunological memory to SARS-CoV-2 assessed for up to 8 months after infection. Science 371, eabf4063 (2021). 10.1126/science.abf4063 33408181 PMC7919858

[7] K. Sauer, T. Harris, An Effective COVID-19 Vaccine Needs to Engage T Cells. Front. Immunol. 11, 581807 (2020). 10.3389/fimmu.2020.581807 33117391 PMC7549399

[8] Y. Peng, A. J. Mentzer, G. Liu, X. Yao, Z. Yin, D. Dong, W. Dejnirattisai, T. Rostron, P. Supasa, C. Liu, C. López-Camacho, J. Slon-Campos, Y. Zhao, D. I. Stuart, G. C. Paesen, J. M. Grimes, A. A. Antson, O. W. Bayfield, D. E. D. P. Hawkins, D. S. Ker, B. Wang, L. Turtle, K. Subramaniam, P. Thomson, P. Zhang, C. Dold, J. Ratcliff, P. Simmonds, T. de Silva, P. Sopp, D. Wellington, U. Rajapaksa, Y. L. Chen, M. Salio, G. Napolitani, W. Paes, P. Borrow, B. M. Kessler, J. W. Fry, N. F. Schwabe, M. G. Semple, J. K. Baillie, S. C. Moore, P. J. M. Openshaw, M. A. Ansari, S. Dunachie, E. Barnes, J. Frater, G. Kerr, P. Goulder, T. Lockett, R. Levin, Y. Zhang, R. Jing, L. P. Ho, R. J. Cornall, C. P. Conlon, P. Klenerman, G. R. Screaton, J. Mongkolsapaya, A. McMichael, J. C. Knight, G. Ogg, T. Dong; Oxford Immunology Network Covid-19 Response T cell Consortium; ISARIC4C Investigators, Broad and strong memory CD4^+^ and CD8^+^ T cells induced by SARS-CoV-2 in UK convalescent individuals following COVID-19. Nat. Immunol. 21, 1336–1345 (2020). 10.1038/s41590-020-0782-6 32887977 PMC7611020

[9] J. Braun, L. Loyal, M. Frentsch, D. Wendisch, P. Georg, F. Kurth, S. Hippenstiel, M. Dingeldey, B. Kruse, F. Fauchere, E. Baysal, M. Mangold, L. Henze, R. Lauster, M. A. Mall, K. Beyer, J. Röhmel, S. Voigt, J. Schmitz, S. Miltenyi, I. Demuth, M. A. Müller, A. Hocke, M. Witzenrath, N. Suttorp, F. Kern, U. Reimer, H. Wenschuh, C. Drosten, V. M. Corman, C. Giesecke-Thiel, L. E. Sander, A. Thiel, SARS-CoV-2-reactive T cells in healthy donors and patients with COVID-19. Nature 587, 270–274 (2020). 10.1038/s41586-020-2598-9 32726801

[10] A. Nelde, T. Bilich, J. S. Heitmann, Y. Maringer, H. R. Salih, M. Roerden, M. Lübke, J. Bauer, J. Rieth, M. Wacker, A. Peter, S. Hörber, B. Traenkle, P. D. Kaiser, U. Rothbauer, M. Becker, D. Junker, G. Krause, M. Strengert, N. Schneiderhan-Marra, M. F. Templin, T. O. Joos, D. J. Kowalewski, V. Stos-Zweifel, M. Fehr, A. Rabsteyn, V. Mirakaj, J. Karbach, E. Jäger, M. Graf, L. C. Gruber, D. Rachfalski, B. Preuß, I. Hagelstein, M. Märklin, T. Bakchoul, C. Gouttefangeas, O. Kohlbacher, R. Klein, S. Stevanović, H. G. Rammensee, J. S. Walz, SARS-CoV-2-derived peptides define heterologous and COVID-19-induced T cell recognition. Nat. Immunol. 22, 74–85 (2021). 10.1038/s41590-020-00808-x 32999467

[11] T. Sekine, A. Perez-Potti, O. Rivera-Ballesteros, K. Strålin, J. B. Gorin, A. Olsson, S. Llewellyn-Lacey, H. Kamal, G. Bogdanovic, S. Muschiol, D. J. Wullimann, T. Kammann, J. Emgård, T. Parrot, E. Folkesson, O. Rooyackers, L. I. Eriksson, J. I. Henter, A. Sönnerborg, T. Allander, J. Albert, M. Nielsen, J. Klingström, S. Gredmark-Russ, N. K. Björkström, J. K. Sandberg, D. A. Price, H. G. Ljunggren, S. Aleman, M. Buggert; Karolinska COVID-19 Study Group, Robust T Cell Immunity in Convalescent Individuals with Asymptomatic or Mild COVID-19. Cell 183, 158–168.e14 (2020). 10.1016/j.cell.2020.08.017 32979941 PMC7427556

[12] I. Schulien, J. Kemming, V. Oberhardt, K. Wild, L. M. Seidel, S. Killmer, F. Sagar, F. Daul, M. Salvat Lago, A. Decker, H. Luxenburger, B. Binder, D. Bettinger, O. Sogukpinar, S. Rieg, M. Panning, D. Huzly, M. Schwemmle, G. Kochs, C. F. Waller, A. Nieters, D. Duerschmied, F. Emmerich, H. E. Mei, A. R. Schulz, S. Llewellyn-Lacey, D. A. Price, T. Boettler, B. Bengsch, R. Thimme, M. Hofmann, C. Neumann-Haefelin, Characterization of pre-existing and induced SARS-CoV-2-specific CD8^+^ T cells. Nat. Med. 27, 78–85 (2021). 10.1038/s41591-020-01143-2 33184509

[13] J. Mateus, A. Grifoni, A. Tarke, J. Sidney, S. I. Ramirez, J. M. Dan, Z. C. Burger, S. A. Rawlings, D. M. Smith, E. Phillips, S. Mallal, M. Lammers, P. Rubiro, L. Quiambao, A. Sutherland, E. D. Yu, R. da Silva Antunes, J. Greenbaum, A. Frazier, A. J. Markmann, L. Premkumar, A. de Silva, B. Peters, S. Crotty, A. Sette, D. Weiskopf, Selective and cross-reactive SARS-CoV-2 T cell epitopes in unexposed humans. Science 370, 89–94 (2020). 10.1126/science.abd3871 32753554 PMC7574914

[14] A. P. Ferretti, T. Kula, Y. Wang, D. M. V. Nguyen, A. Weinheimer, G. S. Dunlap, Q. Xu, N. Nabilsi, C. R. Perullo, A. W. Cristofaro, H. J. Whitton, A. Virbasius, K. J. Olivier Jr., L. R. Buckner, A. T. Alistar, E. D. Whitman, S. A. Bertino, S. Chattopadhyay, G. MacBeath, Unbiased Screens Show CD8^+^ T Cells of COVID-19 Patients Recognize Shared Epitopes in SARS-CoV-2 that Largely Reside outside the Spike Protein. Immunity 53, 1095–1107.e3 (2020). 10.1016/j.immuni.2020.10.006 33128877 PMC7574860

[15] A. Tarke, J. Sidney, C. K. Kidd, J. M. Dan, S. I. Ramirez, E. D. Yu, J. Mateus, R. da Silva Antunes, E. Moore, P. Rubiro, N. Methot, E. Phillips, S. Mallal, A. Frazier, S. A. Rawlings, J. A. Greenbaum, B. Peters, D. M. Smith, S. Crotty, D. Weiskopf, A. Grifoni, A. Sette, Comprehensive analysis of T cell immunodominance and immunoprevalence of SARS-CoV-2 epitopes in COVID-19 cases. Cell Rep Med 2, 100204 (2021). 10.1016/j.xcrm.2021.100204 33521695 PMC7837622

[16] A. Grifoni, D. Weiskopf, S. I. Ramirez, J. Mateus, J. M. Dan, C. R. Moderbacher, S. A. Rawlings, A. Sutherland, L. Premkumar, R. S. Jadi, D. Marrama, A. M. de Silva, A. Frazier, A. F. Carlin, J. A. Greenbaum, B. Peters, F. Krammer, D. M. Smith, S. Crotty, A. Sette, Targets of T Cell Responses to SARS-CoV-2 Coronavirus in Humans with COVID-19 Disease and Unexposed Individuals. Cell 181, 1489–1501.e15 (2020). 10.1016/j.cell.2020.05.015 32473127 PMC7237901

[17] D. Weiskopf, K. S. Schmitz, M. P. Raadsen, A. Grifoni, N. M. A. Okba, H. Endeman, J. P. C. van den Akker, R. Molenkamp, M. P. G. Koopmans, E. C. M. van Gorp, B. L. Haagmans, R. L. de Swart, A. Sette, R. D. de Vries, Phenotype and kinetics of SARS-CoV-2-specific T cells in COVID-19 patients with acute respiratory distress syndrome. Sci. Immunol. 5, eabd2071 (2020). 10.1126/sciimmunol.abd2071 32591408 PMC7319493

[18] T. M. Snyder, R. M. Gittelman, M. Klinger, D. H. May, E. J. Osborne, R. Taniguchi, H. J. Zahid, I. M. Kaplan, J. N. Dines, M. N. Noakes, R. Pandya, X. Chen, S. Elasady, E. Svejnoha, P. Ebert, M. W. Pesesky, P. De Almeida, H. O’Donnell, Q. DeGottardi, G. Keitany, J. Lu, A. Vong, R. Elyanow, P. Fields, J. Greissl, L. Baldo, S. Semprini, C. Cerchione, M. Mazza, O. M. Delmonte, K. Dobbs, G. Carreno-Tarragona, S. Barrio, L. Imberti, A. Sottini, E. Quiros-Roldan, C. Rossi, A. Biondi, L. R. Bettini, M. D’Angio, P. Bonfanti, M. F. Tompkins, C. Alba, C. Dalgard, V. Sambri, G. Martinelli, J. D. Goldman, J. R. Heath, H. C. Su, L. D. Notarangelo, J. Martinez-Lopez, J. M. Carlson, H. S. Robins, Magnitude and Dynamics of the T-Cell Response to SARS-CoV-2 Infection at Both Individual and Population Levels. medRxiv, (2020). 10.1101/2020.07.31.20165647 PMC11747429 39840037

[19] H. Kared, A. D. Redd, E. M. Bloch, T. S. Bonny, H. Sumatoh, F. Kairi, D. Carbajo, B. Abel, E. W. Newell, M. P. Bettinotti, S. E. Benner, E. U. Patel, K. Littlefield, O. Laeyendecker, S. Shoham, D. Sullivan, A. Casadevall, A. Pekosz, A. Nardin, M. Fehlings, A. A. Tobian, T. C. Quinn, SARS-CoV-2-specific CD8+ T cell responses in convalescent COVID-19 individuals. J. Clin. Invest. 131, e145476 (2021). 10.1172/JCI145476 33427749 PMC7919723

[20] S. K. Saini, D. S. Hersby, T. Tamhane, H. R. Povlsen, S. P. Amaya Hernandez, M. Nielsen, A. O. Gang, S. R. Hadrup, SARS-CoV-2 genome-wide T cell epitope mapping reveals immunodominance and substantial CD8^+^ T cell activation in COVID-19 patients. Sci. Immunol. 6, eabf7550 (2021). 10.1126/sciimmunol.abf7550 33853928 PMC8139428

[21] S. Weingarten-Gabbay, S. Klaeger, S. Sarkizova, L. R. Pearlman, D. Y. Chen, K. M. E. Gallagher, M. R. Bauer, H. B. Taylor, W. A. Dunn, C. Tarr, J. Sidney, S. Rachimi, H. L. Conway, K. Katsis, Y. Wang, D. Leistritz-Edwards, M. R. Durkin, C. H. Tomkins-Tinch, Y. Finkel, A. Nachshon, M. Gentili, K. D. Rivera, I. P. Carulli, V. A. Chea, A. Chandrashekar, C. C. Bozkus, M. Carrington, N. Bhardwaj, D. H. Barouch, A. Sette, M. V. Maus, C. M. Rice, K. R. Clauser, D. B. Keskin, D. C. Pregibon, N. Hacohen, S. A. Carr, J. G. Abelin, M. Saeed, P. C. Sabeti; MGH COVID-19 Collection & Processing Team, Profiling SARS-CoV-2 HLA-I peptidome reveals T cell epitopes from out-of-frame ORFs. Cell 184, 3962–3980.e17 (2021). 10.1016/j.cell.2021.05.046 34171305 PMC8173604

[22] G. J. Gorse, G. B. Patel, J. N. Vitale, T. Z. O’Connor, Prevalence of antibodies to four human coronaviruses is lower in nasal secretions than in serum. Clin. Vaccine Immunol. 17, 1875–1880 (2010). 10.1128/CVI.00278-10 20943876 PMC3008199

[23] K. S. MacDonald, K. R. Fowke, J. Kimani, V. A. Dunand, N. J. Nagelkerke, T. B. Ball, J. Oyugi, E. Njagi, L. K. Gaur, R. C. Brunham, J. Wade, M. A. Luscher, P. Krausa, S. Rowland-Jones, E. Ngugi, J. J. Bwayo, F. A. Plummer, Influence of HLA supertypes on susceptibility and resistance to human immunodeficiency virus type 1 infection. J. Infect. Dis. 181, 1581–1589 (2000). 10.1086/315472 10823757

[24] E. E. Ochoa, R. Huda, S. F. Scheibel, J. E. Nichols, D. J. Mock, N. El-Daher, F. M. Domurat, N. J. Roberts Jr., HLA-associated protection of lymphocytes during influenza virus infection. Virol. J. 17, 128 (2020). 10.1186/s12985-020-01406-x 32831108 PMC7444183

[25] D. Ellinghaus, F. Degenhardt, L. Bujanda, M. Buti, A. Albillos, P. Invernizzi, J. Fernández, D. Prati, G. Baselli, R. Asselta, M. M. Grimsrud, C. Milani, F. Aziz, J. Kässens, S. May, M. Wendorff, L. Wienbrandt, F. Uellendahl-Werth, T. Zheng, X. Yi, R. de Pablo, A. G. Chercoles, A. Palom, A. E. Garcia-Fernandez, F. Rodriguez-Frias, A. Zanella, A. Bandera, A. Protti, A. Aghemo, A. Lleo, A. Biondi, A. Caballero-Garralda, A. Gori, A. Tanck, A. Carreras Nolla, A. Latiano, A. L. Fracanzani, A. Peschuck, A. Julià, A. Pesenti, A. Voza, D. Jiménez, B. Mateos, B. Nafria Jimenez, C. Quereda, C. Paccapelo, C. Gassner, C. Angelini, C. Cea, A. Solier, D. Pestaña, E. Muñiz-Diaz, E. Sandoval, E. M. Paraboschi, E. Navas, F. García Sánchez, F. Ceriotti, F. Martinelli-Boneschi, F. Peyvandi, F. Blasi, L. Téllez, A. Blanco-Grau, G. Hemmrich-Stanisak, G. Grasselli, G. Costantino, G. Cardamone, G. Foti, S. Aneli, H. Kurihara, H. ElAbd, I. My, I. Galván-Femenia, J. Martín, J. Erdmann, J. Ferrusquía-Acosta, K. Garcia-Etxebarria, L. Izquierdo-Sanchez, L. R. Bettini, L. Sumoy, L. Terranova, L. Moreira, L. Santoro, L. Scudeller, F. Mesonero, L. Roade, M. C. Rühlemann, M. Schaefer, M. Carrabba, M. Riveiro-Barciela, M. E. Figuera Basso, M. G. Valsecchi, M. Hernandez-Tejero, M. Acosta-Herrera, M. D’Angiò, M. Baldini, M. Cazzaniga, M. Schulzky, M. Cecconi, M. Wittig, M. Ciccarelli, M. Rodríguez-Gandía, M. Bocciolone, M. Miozzo, N. Montano, N. Braun, N. Sacchi, N. Martínez, O. Özer, O. Palmieri, P. Faverio, P. Preatoni, P. Bonfanti, P. Omodei, P. Tentorio, P. Castro, P. M. Rodrigues, A. Blandino Ortiz, R. de Cid, R. Ferrer, R. Gualtierotti, R. Nieto, S. Goerg, S. Badalamenti, S. Marsal, G. Matullo, S. Pelusi, S. Juzenas, S. Aliberti, V. Monzani, V. Moreno, T. Wesse, T. L. Lenz, T. Pumarola, V. Rimoldi, S. Bosari, W. Albrecht, W. Peter, M. Romero-Gómez, M. D’Amato, S. Duga, J. M. Banales, J. R. Hov, T. Folseraas, L. Valenti, A. Franke, T. H. Karlsen; Severe Covid-19 GWAS Group, Genomewide Association Study of Severe Covid-19 with Respiratory Failure. N. Engl. J. Med. 383, 1522–1534 (2020). 10.1056/NEJMoa2020283 32558485 PMC7315890

[26] E. Pairo-Castineira, S. Clohisey, L. Klaric, A. D. Bretherick, K. Rawlik, D. Pasko, S. Walker, N. Parkinson, M. H. Fourman, C. D. Russell, J. Furniss, A. Richmond, E. Gountouna, N. Wrobel, D. Harrison, B. Wang, Y. Wu, A. Meynert, F. Griffiths, W. Oosthuyzen, A. Kousathanas, L. Moutsianas, Z. Yang, R. Zhai, C. Zheng, G. Grimes, R. Beale, J. Millar, B. Shih, S. Keating, M. Zechner, C. Haley, D. J. Porteous, C. Hayward, J. Yang, J. Knight, C. Summers, M. Shankar-Hari, P. Klenerman, L. Turtle, A. Ho, S. C. Moore, C. Hinds, P. Horby, A. Nichol, D. Maslove, L. Ling, D. McAuley, H. Montgomery, T. Walsh, A. C. Pereira, A. Renieri, X. Shen, C. P. Ponting, A. Fawkes, A. Tenesa, M. Caulfield, R. Scott, K. Rowan, L. Murphy, P. J. M. Openshaw, M. G. Semple, A. Law, V. Vitart, J. F. Wilson, J. K. Baillie; GenOMICC Investigators; ISARIC4C Investigators; COVID-19 Human Genetics Initiative; 23andMe Investigators; BRACOVID Investigators; Gen-COVID Investigators, Genetic mechanisms of critical illness in COVID-19. Nature 591, 92–98 (2021). 10.1038/s41586-020-03065-y 33307546

[27] J. R. Habel, T. H. O. Nguyen, C. E. van de Sandt, J. A. Juno, P. Chaurasia, K. Wragg, M. Koutsakos, L. Hensen, X. Jia, B. Chua, W. Zhang, H. X. Tan, K. L. Flanagan, D. L. Doolan, J. Torresi, W. Chen, L. M. Wakim, A. C. Cheng, P. C. Doherty, J. Petersen, J. Rossjohn, A. K. Wheatley, S. J. Kent, L. C. Rowntree, K. Kedzierska, Suboptimal SARS-CoV-2-specific CD8^+^ T cell response associated with the prominent HLA-A*02:01 phenotype. Proc. Natl. Acad. Sci. U.S.A. 117, 24384–24391 (2020). 10.1073/pnas.2015486117 32913053 PMC7533701

[28] M. Shkurnikov, S. Nersisyan, T. Jankevic, A. Galatenko, I. Gordeev, V. Vechorko, A. Tonevitsky, Association of HLA Class I Genotypes With Severity of Coronavirus Disease-19. Front. Immunol. 12, 641900 (2021). 10.3389/fimmu.2021.641900 33732261 PMC7959787

[29] A. Nguyen, J. K. David, S. K. Maden, M. A. Wood, B. R. Weeder, A. Nellore, R. F. Thompson, Human Leukocyte Antigen Susceptibility Map for Severe Acute Respiratory Syndrome Coronavirus 2. J. Virol. 94, ••• (2020). 10.1128/JVI.00510-20 32303592 PMC7307149

[30] Q. Qi, Y. Liu, Y. Cheng, J. Glanville, D. Zhang, J. Y. Lee, R. A. Olshen, C. M. Weyand, S. D. Boyd, J. J. Goronzy, Diversity and clonal selection in the human T-cell repertoire. Proc. Natl. Acad. Sci. U.S.A. 111, 13139–13144 (2014). 10.1073/pnas.1409155111 25157137 PMC4246948

[31] A. S. Shomuradova, M. S. Vagida, S. A. Sheetikov, K. V. Zornikova, D. Kiryukhin, A. Titov, I. O. Peshkova, A. Khmelevskaya, D. V. Dianov, M. Malasheva, A. Shmelev, Y. Serdyuk, D. V. Bagaev, A. Pivnyuk, D. S. Shcherbinin, A. V. Maleeva, N. T. Shakirova, A. Pilunov, D. B. Malko, E. G. Khamaganova, B. Biderman, A. Ivanov, M. Shugay, G. A. Efimov, SARS-CoV-2 Epitopes Are Recognized by a Public and Diverse Repertoire of Human T Cell Receptors. Immunity 53, 1245–1257.e5 (2020). 10.1016/j.immuni.2020.11.004 33326767 PMC7664363

[32] M. Andreatta, M. Nielsen, Gapped sequence alignment using artificial neural networks: Application to the MHC class I system. Bioinformatics 32, 511–517 (2016). 10.1093/bioinformatics/btv639 26515819 PMC6402319

[33] G. Chen, D. Wu, W. Guo, Y. Cao, D. Huang, H. Wang, T. Wang, X. Zhang, H. Chen, H. Yu, X. Zhang, M. Zhang, S. Wu, J. Song, T. Chen, M. Han, S. Li, X. Luo, J. Zhao, Q. Ning, Clinical and immunological features of severe and moderate coronavirus disease 2019. J. Clin. Invest. 130, 2620–2629 (2020). 10.1172/JCI137244 32217835 PMC7190990

[34] S. Jutz, J. Leitner, K. Schmetterer, I. Doel-Perez, O. Majdic, K. Grabmeier-Pfistershammer, W. Paster, J. B. Huppa, P. Steinberger, Assessment of costimulation and coinhibition in a triple parameter T cell reporter line: Simultaneous measurement of NF-κB, NFAT and AP-1. J. Immunol. Methods 430, 10–20 (2016). 10.1016/j.jim.2016.01.007 26780292

[35] P. Dash, A. J. Fiore-Gartland, T. Hertz, G. C. Wang, S. Sharma, A. Souquette, J. C. Crawford, E. B. Clemens, T. H. O. Nguyen, K. Kedzierska, N. L. La Gruta, P. Bradley, P. G. Thomas, Quantifiable predictive features define epitope-specific T cell receptor repertoires. Nature 547, 89–93 (2017). 10.1038/nature22383 28636592 PMC5616171

[36] P. A. Szabo, H. M. Levitin, M. Miron, M. E. Snyder, T. Senda, J. Yuan, Y. L. Cheng, E. C. Bush, P. Dogra, P. Thapa, D. L. Farber, P. A. Sims, Single-cell transcriptomics of human T cells reveals tissue and activation signatures in health and disease. Nat. Commun. 10, 4706 (2019). 10.1038/s41467-019-12464-3 31624246 PMC6797728

[37] G. Monaco, B. Lee, W. Xu, S. Mustafah, Y. Y. Hwang, C. Carré, N. Burdin, L. Visan, M. Ceccarelli, M. Poidinger, A. Zippelius, J. Pedro de Magalhães, A. Larbi, RNA-Seq Signatures Normalized by mRNA Abundance Allow Absolute Deconvolution of Human Immune Cell Types. Cell Rep. 26, 1627–1640.e7 (2019). 10.1016/j.celrep.2019.01.041 30726743 PMC6367568

[38] G. Galletti, G. De Simone, E. M. C. Mazza, S. Puccio, C. Mezzanotte, T. M. Bi, A. N. Davydov, M. Metsger, E. Scamardella, G. Alvisi, F. De Paoli, V. Zanon, A. Scarpa, B. Camisa, F. S. Colombo, A. Anselmo, C. Peano, S. Polletti, D. Mavilio, L. Gattinoni, S. K. Boi, B. A. Youngblood, R. E. Jones, D. M. Baird, E. Gostick, S. Llewellyn-Lacey, K. Ladell, D. A. Price, D. M. Chudakov, E. W. Newell, M. Casucci, E. Lugli, Two subsets of stem-like CD8^+^ memory T cell progenitors with distinct fate commitments in humans. Nat. Immunol. 21, 1552–1562 (2020). 10.1038/s41590-020-0791-5 33046887 PMC7610790

[39] S. P. H. van den Berg, I. N. Pardieck, J. Lanfermeijer, D. Sauce, P. Klenerman, D. van Baarle, R. Arens, The hallmarks of CMV-specific CD8 T-cell differentiation. Med. Microbiol. Immunol. (Berl.) 208, 365–373 (2019). 10.1007/s00430-019-00608-7 30989333 PMC6647465

[40] A. Addetia, K. H. D. Crawford, A. Dingens, H. Zhu, P. Roychoudhury, M. L. Huang, K. R. Jerome, J. D. Bloom, A. L. Greninger, Neutralizing Antibodies Correlate with Protection from SARS-CoV-2 in Humans during a Fishery Vessel Outbreak with a High Attack Rate. J. Clin. Microbiol. 58, ••• (2020). 10.1128/JCM.02107-20 32826322 PMC7587101

[41] A. Fontanet, S. Cauchemez, COVID-19 herd immunity: Where are we? Nat. Rev. Immunol. 20, 583–584 (2020). 10.1038/s41577-020-00451-5 32908300 PMC7480627

[42] C. Liang, E. Bencurova, E. Psota, P. Neurgaonkar, M. Prelog, C. Scheller, T. Dandekar, Population-Predicted MHC Class II Epitope Presentation of SARS-CoV-2 Structural Proteins Correlates to the Case Fatality Rates of COVID-19 in Different Countries. Int. J. Mol. Sci. 22, 2630 (2021). 10.3390/ijms22052630 33807854 PMC7961590

[43] A. Anzurez, I. Naka, S. Miki, K. Nakayama-Hosoya, M. Isshiki, Y. Watanabe, M. Nakamura-Hoshi, S. Seki, T. Matsumura, T. Takano, T. Onodera, Y. Adachi, S. Moriyama, K. Terahara, N. Tachikawa, Y. Yoshimura, H. Sasaki, H. Horiuchi, N. Miyata, K. Miyazaki, M. Koga, K. Ikeuchi, H. Nagai, M. Saito, E. Adachi, H. Yotsuyanagi, S. Kutsuna, A. Kawashima, Y. Miyazato, N. Kinoshita, C. Kouno, K. Tanaka, Y. Takahashi, T. Suzuki, T. Matano, J. Ohashi, A. Kawana-Tachikawa, Association of HLA-DRB1*09:01 with severe COVID-19. HLA 98, 37–42 (2021). 10.1111/tan.14256 33734601 PMC8251239

[44] P. Correale, L. Mutti, F. Pentimalli, G. Baglio, R. E. Saladino, P. Sileri, A. Giordano, HLA-B*44 and C*01 Prevalence Correlates with Covid19 Spreading across Italy. Int. J. Mol. Sci. 21, 5205 (2020). 10.3390/ijms21155205 32717807 PMC7432860

[45] V. Venturi, M. F. Quigley, H. Y. Greenaway, P. C. Ng, Z. S. Ende, T. McIntosh, T. E. Asher, J. R. Almeida, S. Levy, D. A. Price, M. P. Davenport, D. C. Douek, A mechanism for TCR sharing between T cell subsets and individuals revealed by pyrosequencing. J. Immunol. 186, 4285–4294 (2011). 10.4049/jimmunol.1003898 21383244

[46] H. Y. Zheng, M. Zhang, C. X. Yang, N. Zhang, X. C. Wang, X. P. Yang, X. Q. Dong, Y. T. Zheng, Elevated exhaustion levels and reduced functional diversity of T cells in peripheral blood may predict severe progression in COVID-19 patients. Cell. Mol. Immunol. 17, 541–543 (2020). 10.1038/s41423-020-0401-3 32203186 PMC7091621

[47] B. Diao, C. Wang, Y. Tan, X. Chen, Y. Liu, L. Ning, L. Chen, M. Li, Y. Liu, G. Wang, Z. Yuan, Z. Feng, Y. Zhang, Y. Wu, Y. Chen, Reduction and Functional Exhaustion of T Cells in Patients With Coronavirus Disease 2019 (COVID-19). Front. Immunol. 11, 827 (2020). 10.3389/fimmu.2020.00827 32425950 PMC7205903

[48] E. L. Brown, H. T. Essigmann, Original Antigenic Sin: The Downside of Immunological Memory and Implications for COVID-19. MSphere 6, e00056-21 (2021). 10.1128/mSphere.00056-21 33692194 PMC8546681

[49] J. C. Law, W. H. Koh, P. Budylowski, J. Lin, F. Yue, K. T. Abe, B. Rathod, M. Girard, Z. Li, J. M. Rini, S. Mubareka, A. McGeer, A. K. Chan, A. C. Gingras, T. H. Watts, M. A Ostrowski, Systematic Examination of Antigen-Specific Recall T Cell Responses to SARS-CoV-2 versus Influenza Virus Reveals a Distinct Inflammatory Profile. J. Immunol. 206, 37–50 (2021). 10.4049/jimmunol.2001067 33208459 PMC7750861

[50] Z. M. Ndhlovu, S. W. Kazer, T. Nkosi, F. Ogunshola, D. M. Muema, G. Anmole, S. A. Swann, A. Moodley, K. Dong, T. Reddy, M. A. Brockman, A. K. Shalek, T. Ndung’u, B. D. Walker, Augmentation of HIV-specific T cell function by immediate treatment of hyperacute HIV-1 infection. Sci. Transl. Med. 11, eaau0528 (2019). 10.1126/scitranslmed.aau0528 31118290 PMC6901350

[51] H. L. Oh, A. Chia, C. X. Chang, H. N. Leong, K. L. Ling, G. M. Grotenbreg, A. J. Gehring, Y. J. Tan, A. Bertoletti, Engineering T cells specific for a dominant severe acute respiratory syndrome coronavirus CD8 T cell epitope. J. Virol. 85, 10464–10471 (2011). 10.1128/JVI.05039-11 21813600 PMC3187484

[52] K. M. Campbell, G. Steiner, D. K. Wells, A. Ribas, A. Kalbasi, Prediction of SARS-CoV-2 epitopes across 9360 HLA class I alleles. bioRxiv, (2020).

[53] A. Grifoni, J. Sidney, Y. Zhang, R. H. Scheuermann, B. Peters, A. Sette, A Sequence Homology and Bioinformatic Approach Can Predict Candidate Targets for Immune Responses to SARS-CoV-2. Cell Host Microbe 27, 671–680.e2 (2020). 10.1016/j.chom.2020.03.002 32183941 PMC7142693

[54] J. D. Altman, M. M. Davis, MHC-Peptide Tetramers to Visualize Antigen-Specific T Cells. Curr. Protoc. Immunol. 115, 3.1, 44 (2016). 10.1002/cpim.14 27801510

[55] I. Chen, B. M. Dorr, D. R. Liu, A general strategy for the evolution of bond-forming enzymes using yeast display. Proc. Natl. Acad. Sci. U.S.A. 108, 11399–11404 (2011). 10.1073/pnas.1101046108 21697512 PMC3136257

[56] M. W. Popp, J. M. Antos, H. L. Ploegh, Site-specific protein labeling via sortase-mediated transpeptidation. *Curr Protoc Protein Sci* **Chapter 15**, Unit 15 13 (2009). 10.1002/0471140864.ps1503s56 PMC5551486 19365788

[57] F. A. Wolf, P. Angerer, F. J. Theis, SCANPY: Large-scale single-cell gene expression data analysis. Genome Biol. 19, 15 (2018). 10.1186/s13059-017-1382-0 29409532 PMC5802054

[58] B. Hie, B. Bryson, B. Berger, Efficient integration of heterogeneous single-cell transcriptomes using Scanorama. Nat. Biotechnol. 37, 685–691 (2019). 10.1038/s41587-019-0113-3 31061482 PMC6551256

[59] D. Aran, A. P. Looney, L. Liu, E. Wu, V. Fong, A. Hsu, S. Chak, R. P. Naikawadi, P. J. Wolters, A. R. Abate, A. J. Butte, M. Bhattacharya, Reference-based analysis of lung single-cell sequencing reveals a transitional profibrotic macrophage. Nat. Immunol. 20, 163–172 (2019). 10.1038/s41590-018-0276-y 30643263 PMC6340744

[60] D. van Dijk, R. Sharma, J. Nainys, K. Yim, P. Kathail, A. J. Carr, C. Burdziak, K. R. Moon, C. L. Chaffer, D. Pattabiraman, B. Bierie, L. Mazutis, G. Wolf, S. Krishnaswamy, D. Pe’er, Recovering Gene Interactions from Single-Cell Data Using Data Diffusion. Cell 174, 716–729.e27 (2018). 10.1016/j.cell.2018.05.061 29961576 PMC6771278

