# Supplementary material for: Allelic variation in class I HLA determines CD8+ T cell repertoire shape and cross-reactive memory responses to SARS-CoV-2
Source: Sci Immunol. 2021 Nov 18:eabk3070. doi: 10.1126/sciimmunol.abk3070 (PMC9017864; doi:10.1126/sciimmunol.abk3070)
Supplement: Supplementary file 1 — Figs. S1 to S8 Tables S1 and S2 [file sciimmunol.abk3070_sm.pdf]

Science Immunology
AAAS

Supplementary Materials for

Allelic variation in class I HLA determines CD8^{+} T cell repertoire shape and cross-reactive memory responses to SARS-CoV-2

Joshua M. Francis *et al.*

Corresponding author: Daniel C. Pregibon, dpregibon@repertoire.com

DOI: 10.1126/sciimmunol.abk3070

The PDF file includes:

Figs. S1 to S8
Tables S1 and S2

Other Supplementary Material for this manuscript includes the following:

Data files S1 to S9

Supplementary Materials for

Allelic variation in Class I HLA determines $CD8^+$ T cell repertoire shape and cross-reactive memory responses to SARS-CoV-2

Joshua M. Francis[1], Del Leistritz-Edwards[1], Augustine Dunn[1], Christina Tarr[1], Jesse Lehman[1], Conor Dempsey[1], Andrew Hamel[1], Violeta Rayon[1], Gang Liu[1], Yuntong Wang[1], Marcos Wille[1], Melissa Durkin[1], Kane Hadley[1], Aswathy Sheena[1], Benjamin Roscoe[1], Mark Ng[1], Graham Rockwell[1], Margaret Manto[1], Elizabeth Gienger[1], Joshua Nickerson[1], MGH COVID-19 Collection and Processing Team[2], Amir Moarefi[1], Michael Noble[1], Thomas Malia[1], Philip D. Bardwell[1], William Gordon[1], Joanna Swain[1], Mojca Skoberne[1], Karsten Sauer[1], Tim Harris[1], Ananda W. Goldrath[3], Alex K. Shalek[1,4,5,6], Anthony J. Coyle[1], Christophe Benoist[7], Daniel C. Pregibon[1*]

Correspondence to: dpregibon@repertoire.com

This PDF file includes:

Figs. S1 to S8
Tables S1 and S2
Captions for Data files S1 to S9

Other Supplementary Materials for this manuscript include the following:

Data files S1 to S9 (.xlsx)

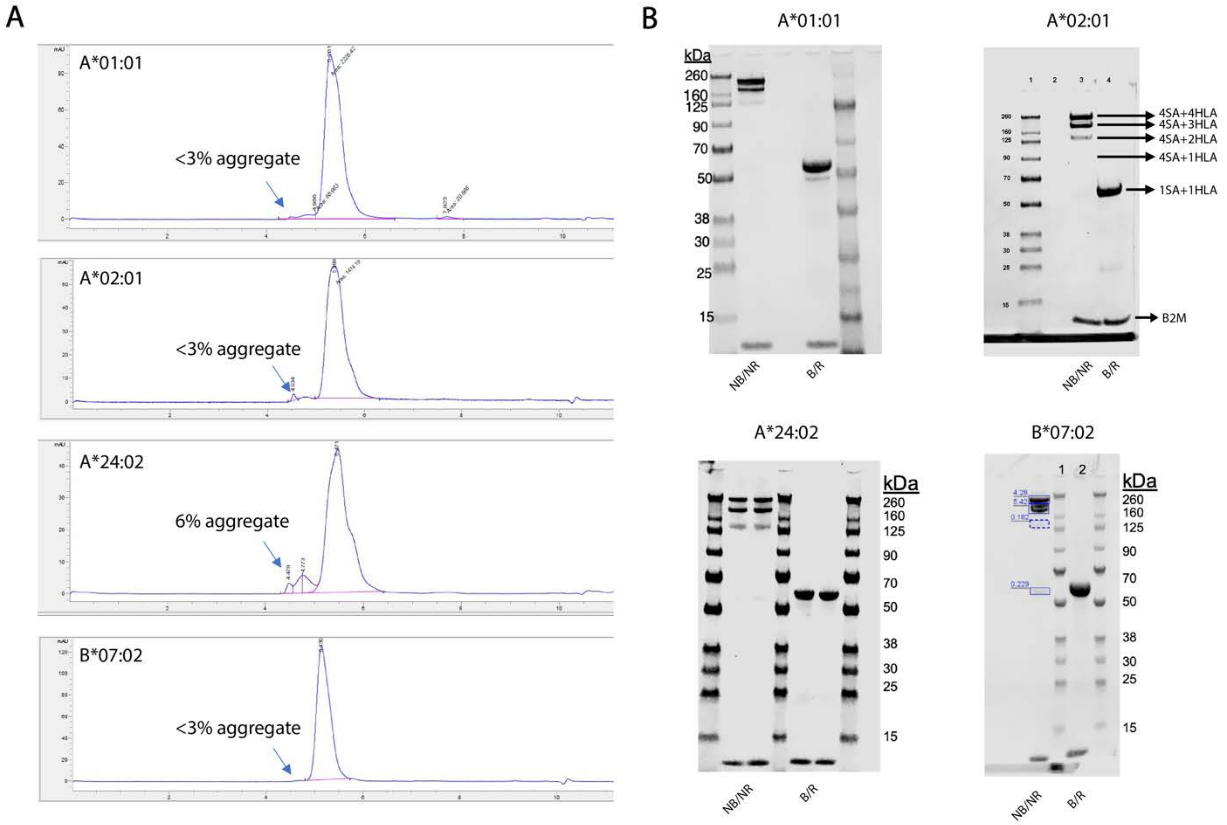

Fig. S1.

Quality assessment of placeholder peptide-loaded HLA tetramers. Assessment by **(A)** analytical SEC and **(B)** SDS-PAGE analysis show a highly multimeric structures with little aggregation. **(B)** SDS-PAGE analysis of placeholder peptide-loaded HLA tetramers either boiled and reduced (B/R), or non-boiled and non-reduced (NB/NR), in which the streptavidin quaternary structure is maintained. Densitometry analysis indicates all preparations are >80% trimer/tetramer fraction.

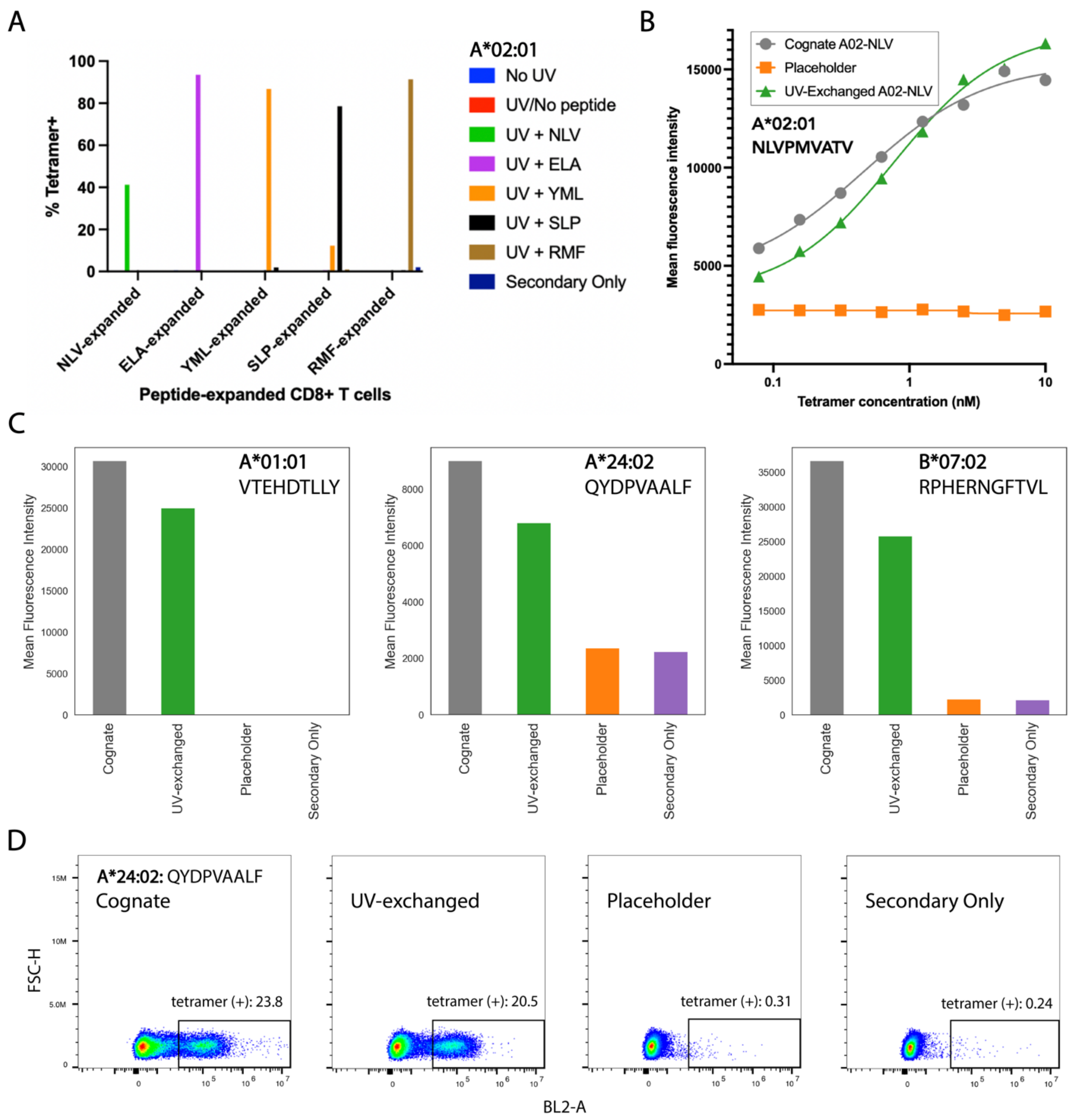

Fig. S2.

Assessment of UV-mediated peptide-exchange tetramers. (A) Cell binding with peptide-exchanged HLA tetramers on corresponding peptide-expanded, healthy donor T cells. Placeholder-loaded HLA tetramers ("No UV") were UV-exchanged as indicated in the **Methods**, either in the presence ("UV + PEP") or absence ("UV/No peptide") of rescue peptide, and used to stain the indicated peptide-expanded cells. Peptide abbreviations and sources: NLVPMVATV (NLV, CMV pp65), ELAGIGILTV (ELA, MART-1), YMLDLQPETT (YML, HPV16 E7), SLPITVYYA (SLP, HSV gD1), RMFPNAPYL (RMF, Wilms tumor antigen 1), VTEHDTLLY (CMV pp50), QYDPVAALF (CMV pp65), RPHERNGFTVL (CMV pp65). **(B-D)** Comparison of cell staining with "industry standard" biotin-mediated tetramers that were produced by

refolding with a cognate peptide (“Cognate”), placeholder-loaded covalent tetramers (“Placeholder”), placeholder-to-cognate UV-exchanged covalent tetramers (“UV-Exchanged”), or secondary antibody only (“Secondary Only”). **(B)** Mean fluorescence intensity (MFI) of NLV-expanded cells stained with titrations of A*02:01 tetramers. **(C)** Comparison of MFI of peptide-expanded cells stained with corresponding A*01:01, A*24:02 and B*07:02 tetramers at 20nM. **(D)** Cytometry scatter plots corresponding to the A*24:02 data represented in panel C, comparing the staining of cells expanded with peptide QYDPVAALF and incubated with biotin-mediated or covalent, UV-exchanged tetramers.

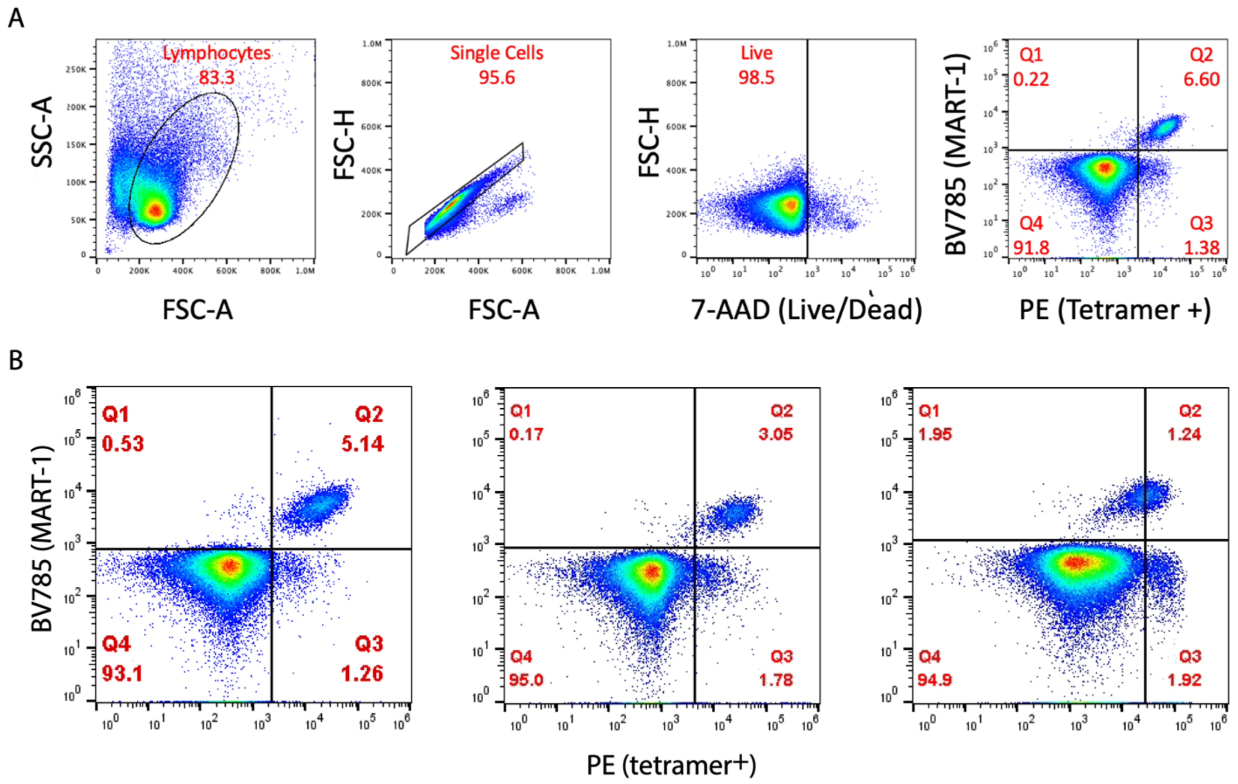

Fig S3.

Gating strategy for cell sorting prior to single-cell analysis. (A) Cells were sorted for lymphocytes, singlets, live fraction, and tetramer positivity. Anti-MART-1 cells expanded with the peptide ELAGIGILTV, labeled with anti-CD8 BV785, and stained with an ELAGIGILTV-A*02:01 tetramer were sorted in parallel to subject T cells stained with a B*07:02, SARS-CoV-2 tetramer library. In the scatter plot on the right, the B*07:02 tetramer (+) cells from the subject sample are shown in quadrant 3 (Q3) and tetramer (+) anti-MART-1 cells are shown in quadrant 2 (Q2). **(B)** Representative sorts of cells stained with A*01:01 (left), A*02:01 (middle), and A*24:02 (right) tetramer libraries.

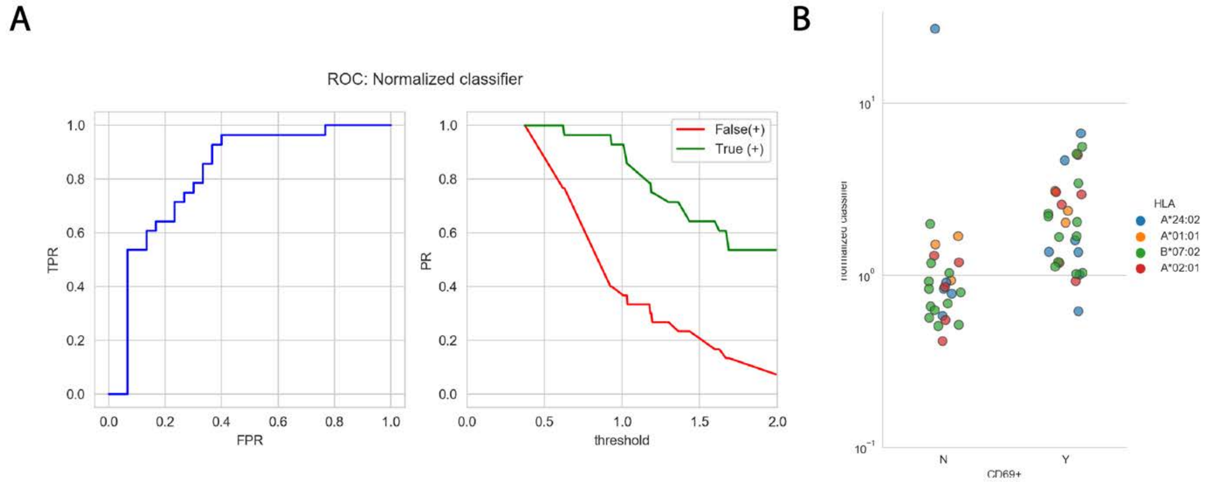

Fig. S4.

Receiver-operator analysis for TCR-peptide-HLA hit identification. True positives were determined by CD69 expression in recombinant TCR assays, read out by flow. **(A)** Receiver-operator curve showing true positive rate (TPR) versus false positive rate (FPR), and associated TPR (green) and FPR (red) versus classifier threshold. **(B)** Shown are distributions of classifiers for interactions found to be negative (N) by CD69 assay or positive (Y).

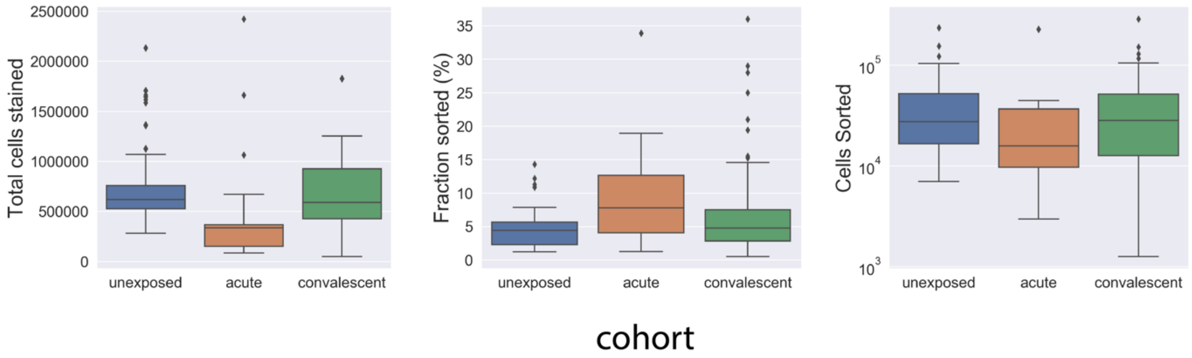

Fig. S5.

CD8⁺ T cells interrogated and sorted by cohort for all samples used in this study. Shown are distributions of cell counts representing the number of viable cells recovered after CD8 enrichment and subsequently stained with tetramer libraries (left), fraction of cells sorted after staining (center), and number of cells sorted prior to single-cell analysis (right). Median cells sorted were 27,500, 15,800, and 28,200 for unexposed, acute, and convalescent, respectively. Of these, 15,000 per sample were targeted for single-cell encapsulation and sequencing.

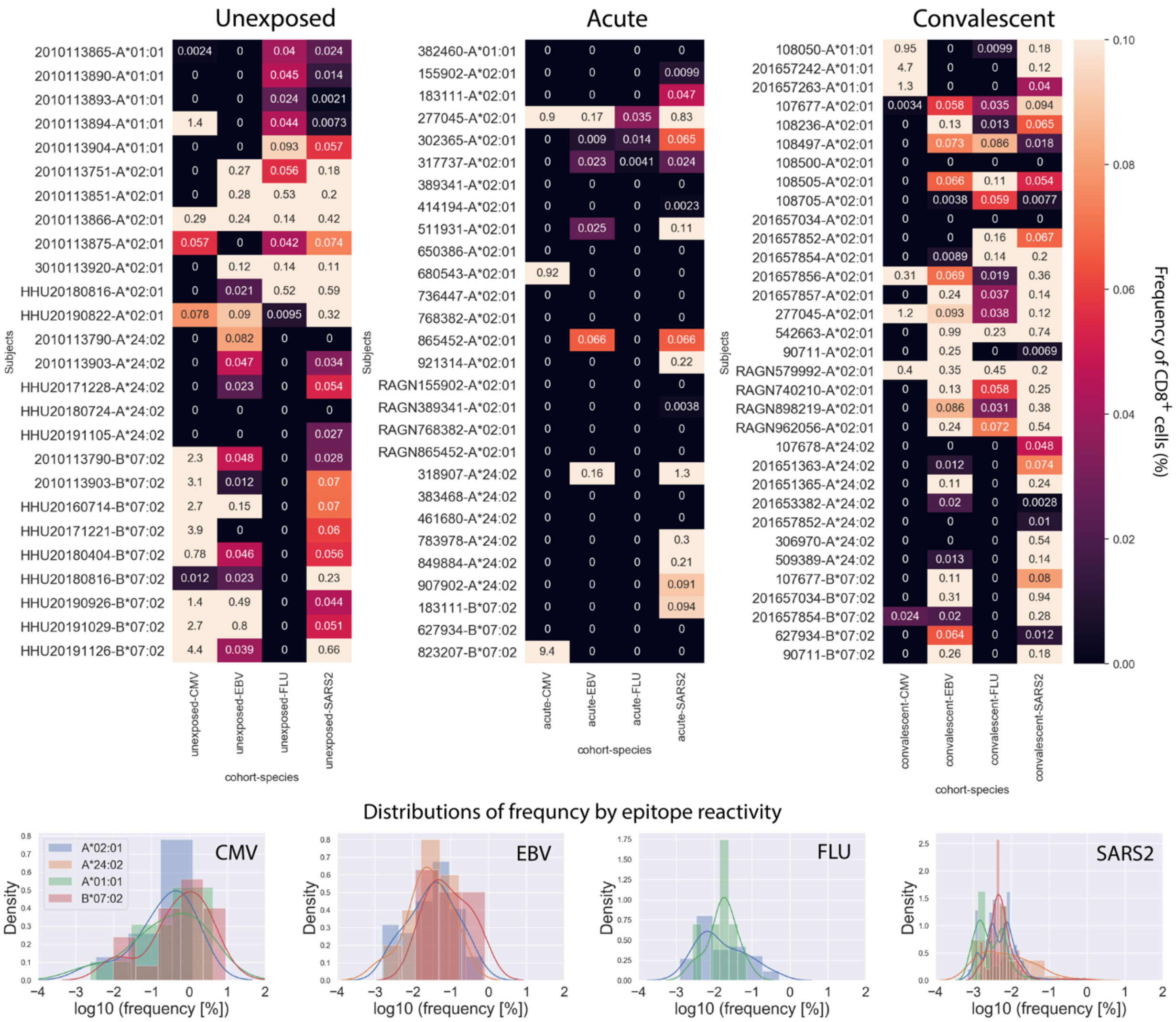

Fig. S6.

Cumulative reactivity to CMV, EBV, influenza, and SARS-CoV-2 (SARS2) by subject, cohort, and HLA. Shown are heatmaps of cumulative frequencies (sum of calculated frequencies as described in **Methods**) of T cells found to be reactive to any epitope assayed within the designated species for each cohort. Shown in the bottom panel are distributions of cumulative frequencies by epitope and HLA for each species across all cohorts.

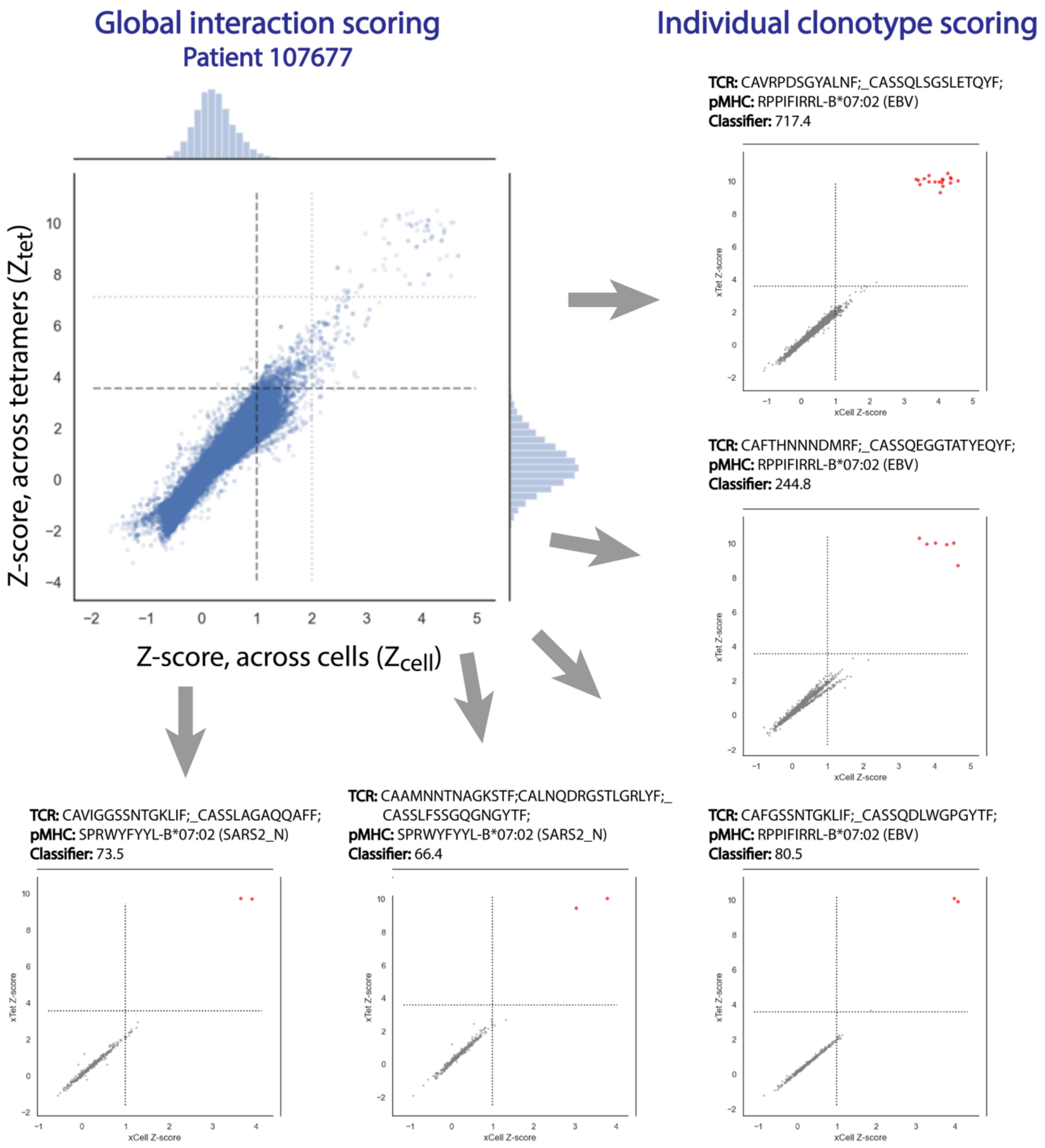

Fig. S7.

Putative hit identification. Z-scores for each cell-pMHC interaction are calculated across all cells and all tetramers and plotted on the x-axis and y-axis, respectively. A classifier score for each clonotype (possibly multiple cells) is calculated using a product of both Z-scores (each averaged across cells within clonotype) and the number of cells represented by the clonotype. Shown is a representative analysis for a convalescent subject sample stained with the B*07:02 tetramer library, including a global analysis across cells and several individual clonotypes

isolated from the global plot for epitopes RPPIFIRRL and SPRWYFYYL (shown in red in respective plots).

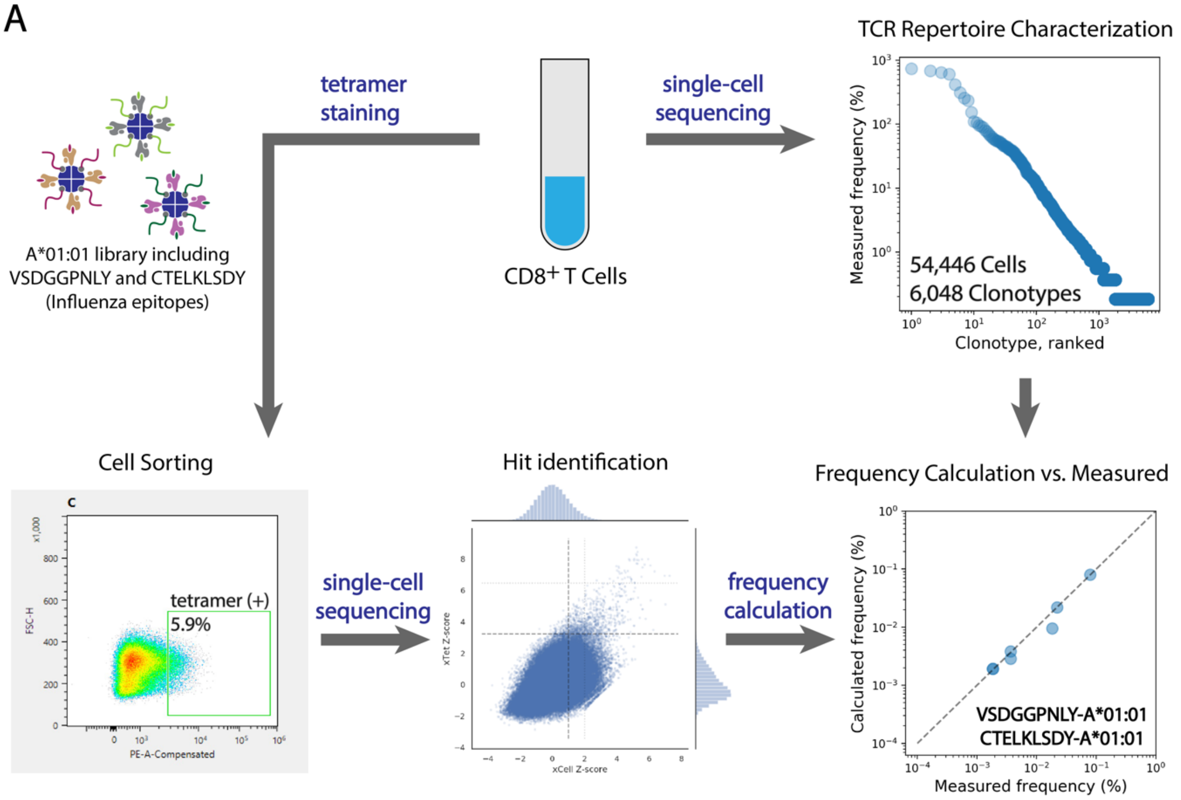

B

clonotype	epitope	antigen	measured	calculated	fold difference
CAASETSGTYKYIF;_CASSLDGQGPLYGYTF;	VSDGGPNLY-A*01:01	FLUA_PB1	0.078977	0.079860	0.988950
CAAAPTSGTYKYIF;_CASSPDGQAPNYGYTF;	VSDGGPNLY-A*01:01	FLUA_PB1	0.022040	0.022130	0.995950
CAVNKGYGNKLVF;_CASSERLAGINNEQFF;	VSDGGPNLY-A*01:01	FLUA_PB1	0.018367	0.009622	1.908904
CVVALDNYGQNFVF;_CATQDITGNYGYTF;	VSDGGPNLY-A*01:01	FLUA_PB1	0.003673	0.003849	0.954452
CVVSGDKVIF;_CASSLGAGSLSSYNEQFF;	CTELKLSDY-A*01:01	FLUA_NP	0.003673	0.002886	1.272603
CAASATSGTYKYIF;_CASSLDGQGPLYGYTF;	VSDGGPNLY-A*01:01	FLUA_PB1	0.001837	0.001924	0.954452
CVGYNAGNMLTF;_CASSGSSWEKETQYF;	CTELKLSDY-A*01:01	FLUA_NP	NaN	0.001924	NaN
CVVSTDKLIF;_CASSPGTDFSYEQYF;	CTELKLSDY-A*01:01	FLUA_NP	0.001837	0.001924	0.954452
No-alpha-detected;_CASSLDGQGPLYGYTF;	VSDGGPNLY-A*01:01	FLUA_PB1	NaN	0.001924	NaN

Fig. S8.
Assessment of frequency calculation for Influenza A specific CD8$^+$ T cells in a well-characterized sample. (A) Multiclonal T cells were characterized by direct encapsulation and single-cell sequencing to determine frequency of TCR clonotypes. In parallel, these cells were stained with an A*01:01 tetramer library containing two epitopes for Influenza A, tetramer positive cells were sorted, and reactivities were determined using the approach described in the **Methods** section. Frequencies of the parent sample were calculated by taking the product of the fraction of the reactive clonotype in the sorted sample and fraction of tetramer (+) cells sorted. **(B)** Table of putative hits quantified in sorted (calculated, %) and unsorted (measured, %) samples.

Table S1.

Putative hits identified by antigen, representing the number of clonotypes that were found to be specific to any epitope within the given antigen.

antigen	A*01:01	A*02:01	A*24:02	B*07:02	sum
SARS2_ORF1AB	99	419	77	26	621
SARS2_SPIKE	14	158	46	6	224
SARS2_N	1	45		72	118
SARS2_3A	10	73	6	1	90
SARS2_M	7	24	11	2	44
SARS2_E	4	10			14
SARS2_7A		12		1	13
SARS2_9B	1	9			10
SARS2_10		6	1		7
SARS2_14		6			6
SARS2_7B		5			5
SARS2_8		5			5
SARS2_6	1	3			4

Table S2.

Gene sets used for single-cell transcriptomics analysis.

set	gene
naive	IL7R; SELL; TCF7; CCR7; LEF1; CD28; LRRN3
memory	GZMK; AQP3; LMNA; ID3; EPHA4; GPR15
effector	NKG7; CST7; GZMA; GNLY; GZMH; GZMB; PRF1; KLRG1; CCL4; RUNX3; ITGAL; KLRB1; IL2RB; PRDM1; HLA-DQB1; FAS; CD58; EOMES; IFNG; TBX21; TNF; CCL3; FASLG
exhaustion	TIGIT; LAG3; TOX; PDCD1; HAVCR2; CTLA4
discriminators	LTB; CD69; CD44; CD27; TMEM123; CXCR3; CD8A; CD8B; CD4

Data file S1.

Raw data from manuscript figures.

Data file S2.

Subject summary and metadata.

Data file S3.

Putative TCR-peptide-HLA hits identified in this study.

Data file S4.

Summary of SARS-CoV-2 epitopes identified in this study compared to those obtained in other publications. The ranking of epitopes is a qualitative measure of relevance by HLA, calculated using the number of clonotypes (Francis), frequency of positive response (Tarke and Feretti), and p-value (Saini).

Data file S5.

Epitopes and hits discovered in this work that would be impacted by high-threat SARS-CoV-2 variants. Shown are the epitopes included in this study that align with the specified mutation along with the number of clonotypes found to bind that epitope across the specified number of cells.

Data file S6.

Recombinant TCR-peptide-HLA interactions confirmed by functional testing. Shown are the cohort, patient identifier, HLA, epitope, antigen, and alpha/beta CDR3 sequences for interactions originally identified across nCells as well as percentage of TCR transduced cells found to be

CD69+ or expressing NFAT after stimulation. Thresholds for positive interactions were 20% positive and 3-fold over DMSO for CD69 expression and NFAT signaling, respectively.

Data file S7.

Representation of transcriptional phenotype by epitope specificity and cohort. These values are consistent with the cells/clusters represented in Figure 5 of the main text.

Data file S8.

Composition of tetramer libraries indicating HLA allele, peptide sequence, and DNA barcode.

Data file S9.

TCR sequences for anti-MART-1 T cells used in sample multiplexing. Sequences annotated indicate the top clonotypes (representing ~99%) of a cell line expanded using peptide ELAGIGILTV in A*02:01.
